# The effectiveness and cost-effectiveness of integrating mental health services in primary care in low- and middle-income countries: systematic review

**DOI:** 10.1192/bjb.2020.35

**Published:** 2021-02

**Authors:** Leonardo Cubillos, Sophia M. Bartels, William C. Torrey, John Naslund, José Miguel Uribe-Restrepo, Chelsea Gaviola, Sergio Castro Díaz, Deepak T. John, Makeda J. Williams, Magda Cepeda, Carlos Gómez-Restrepo, Lisa A. Marsch

**Affiliations:** 1 Center for Technology and Behavioral Health, Geisel School of Medicine at Dartmouth, USA; 2 Department of Psychiatry, Geisel School of Medicine at Dartmouth, USA; 3 The Dartmouth Institute, Geisel School of Medicine at Dartmouth, USA; 4 Department of Global Health and Social Medicine, Harvard Medical School, USA; 5 Department of Psychiatry and Mental Health, Pontificia Universidad Javeriana, Colombia; 6 Department of Clinical Epidemiology and Biostatistics, Pontificia Universidad Javeriana, Colombia; 7 Center for Global Mental Health Research, National Institute of Mental Health, USA

**Keywords:** Mental health integration, depression, alcohol use, primary care, provision of services

## Abstract

**Aims and method:**

This systematic review examines the effectiveness and cost-effectiveness of behavioural health integration into primary healthcare in the management of depression and unhealthy alcohol use in low- and middle-income countries. Following PRISMA guidelines, this review included research that studied patients aged ≥18 years with unhealthy alcohol use and/or depression of any clinical severity. An exploration of the models of integration was used to characterise a typology of behavioural health integration specific for low- and middle-income countries.

**Results:**

Fifty-eight articles met inclusion criteria. Studies evidenced increased effectiveness of integrated care over treatment as usual for both conditions. The economic evaluations found increased direct health costs but cost-effective estimates. The included studies used six distinct behavioural health integration models.

**Clinical implications:**

Behavioural health integration may yield improved health outcomes, although it may require additional resources. The proposed typology can assist decision-makers to advance the implementation of integrated models.

## Burden of depression and unhealthy alcohol use

Depression and unhealthy alcohol use are worldwide public health problems. Depression is the single most significant contributor to global disability, accounting for 7.5% of all years lived with disability.^1^ Unhealthy alcohol use accounts for 5.9% of all global deaths and 5.1% of the entire global burden of disease,^2^ and >80% of this occurs in low- and middle-income countries. Individuals suffering from depression and unhealthy alcohol use are at increased risk for poorer health outcomes from other illnesses, such as tuberculosis, maternal and infant conditions and HIV/AIDS.^3,4^ In low- and middle-income countries, societal impacts related to depression and unhealthy alcohol use can exceed healthcare costs.^5,6^ The affected populations have higher rates of unemployment, reduced job functioning and lower educational attainment.^7^

Despite the existence of cost-effective interventions for these conditions, up to 90% of individuals living with mental illnesses in low- and middle-income countries lack access to care.^8,9^ Moreover, in low- and low-middle-income countries, only 1% of the population with substance use disorders has access to minimally adequate treatment.^10^ Factors like low levels of public expenditures, reliance on out-of-pocket payments, preferential funding of hospital-based models of care and significant workforce shortages reduce the availability and accessibility of mental healthcare.^11–13^

## Typology of behavioural health integration in high-income countries

Behavioural health integration into primary care is thought to be an effective way to reduce the treatment gap in resource-constrained settings.^14^ Nonetheless, the vast majority of studies assessing the effectiveness of this integration originate in high-income countries. A meta-analysis, including mostly studies from high-income countries, found moderate-quality evidence that brief interventions in primary healthcare can reduce alcohol consumption in unhealthy drinkers compared with minimal or no intervention.^15^ Similarly, research studies from high-income countries revealed significantly better outcomes for adults with depression treated with collaborative care management compared with care as usual.^16,17^

In high-income countries, different integration models have been classified based on the nature and level of coordination between highly specialised providers. Several high-income countries have developed their own classification adapted to their health system, and these typologies are commonly used in research and healthcare management. For example, the United States Substance Abuse and Mental Health Services Administration conceptualised a typology based on the degree of collaboration between primary care and behavioural healthcare specialists.^18^ At the most basic level, specialists refer patients to another location (coordinated care). At the intermediate level, providers deliver care at the same site but do not share treatment plans (colocated care). At the highest level of integration, specialists are part of the same team with a unique treatment plan, and the patient experiences a single system (integrated care). Collaborative care management, the most commonly studied integration models in high-income countries,^19^ is most often located in this highest level of integration. However, we are unaware of the existence of a typology built specifically for low- and middle-income countries.

## Behavioural health integration in low- and middle-income countries

In low- and middle-income countries, researchers have studied task-shifting, often referred as task-sharing, which is the use of non-specialist primary health workers (such as general practitioners or nurses) and lay health workers to deliver mental health interventions and increase the availability of mental healthcare services.^20–22^ A review of evidence found that interventions delivered by lay health workers may increase the number of adults recovering from depression, reduce symptoms for mothers with perinatal depression and decrease the quantity of alcohol consumed by unhealthy drinkers.^23^ In another review, Seidman and Atun^24^ found task-shifting to hold potential for cost-saving and efficiency improvements in health systems in the care of tuberculosis and HIV/AIDS. Evidence for mental health conditions is unclear.^24^ Both reviews found minimal relevant economic studies.

This systematic review aims to summarise the evidence of effectiveness and economic evaluation of the behavioural health integration of the management of depression and unhealthy alcohol use into primary healthcare in low- and middle-income countries. For the purpose of this review, all types of economic evaluations (such as cost-minimisation, cost–utility, cost–benefit and cost-effectiveness analysis) were included.^25^ We included all types and levels of severity of depression and unhealthy alcohol use. Additionally, we propose a typology to characterise the models of behavioural health integration in low- and middle-income countries, using the evidence of the experimental studies included in the review. This typology could assist hospital and district managers, programme planners and policy makers in their decisions to improve the availability of mental healthcare services.

This systematic review is part of the formative phase of Project Detection and Integrated Care for Depression and Alcohol Use in Primary Care (DIADA), an implementation research project in Colombia, Peru and Chile, funded by the National Institute of Mental Health. Project DIADA studies technology-enhanced service delivery models for treating comorbid depression and unhealthy alcohol use in primary healthcare in multiple sites in urban and rural Colombia.^26^

## Method

### Protocol and registration

We designed this systematic review according to Preferred Reporting Items for Systematic Reviews and Meta-Analyses guidelines.^27^ We registered this review in the PROSPERO International Registry of Systematic Reviews (identifier CRD42017057340).

### Phase 1: search strategy

This systematic review had five phases. In the first phase, medical librarians developed the search strategy, translating research concepts into controlled subject headings and natural language terms. The search included articles from 1990 to 2017. We chose to start the search at 1990 because before this date few, if any, studies in low- and middle-income countries were utilising behavioural health integration models. The following databases were searched for relevant abstracts: Medline – PubMed (search date 28 April 2017), Web of Science (search date 28 April 2017), PsycINFO (search date 28 April 2017), EMBASE (search date 4 May 2017), Cochrane Central Register of Controlled Trials (search date 28 April 2017) and the World Health Organization's (WHO) Global Index Medicus (search date 28 April 2017). The search also included relevant conference proceedings, grey literature and review references in related articles (Table 1). Abstracts in English, French, Spanish and Portuguese were included based on co-authors’ proficiency in these languages. The search found 8786 abstracts after removing duplicates. Search keywords included, but were not limited to, ‘depression’, ‘alcohol use disorder’, ‘integrated care’ and ‘developing country’. A full list of search terms for all databases searched, including PubMed (Medline), can be found under Supplementary File 1 available at https://doi.org/10.1192/bjb.2020.35.

**Table 1:** Overview of databases searched

Database	Platform	Years covered	Date conducted	No. of results
Medline	PubMed	1990 to current	28 April 2017	2520
EMBASE	Elsevier	1990 to current	4 May 2017	2927
Web of Science	Thomson Reuters	1990 to current	28 April 2017	5181
Cochrane Central Register of Controlled Trials	Wiley	1990 to current DSR: issue 4, April 2017 Trials: issue 3, March 2017 Methods: issue 3, July 2012 EconEval: issue 2, 2017	28 April 2017	376 (DSR: 31 Trials: 339 Methods: 2 EconEval: 4)
WHO Global Index Medicus	globalhealthlibrary.net	1990 to current	28 April 2017	1254 (LILACS: 775 WPRIM: 356 IMEMR: 61 IMSEAR: 53 AIM: 9)
PsycINFO	ProQuest	1990 to current	28 April 2017	1241
Total				13 499
Total with duplicates removed				8786

WHO Global Index Medicus search did not include low- and middle-income countries concept. DSR, Database of Systematic Reviews; EconEval, economic evaluations; WHO, World Health Organization; LILACS, Latin American & Caribbean Health Science Literature; WPRIM, Western Pacific Region Index Medicus; IMEMR, Index Medicus for the Eastern Mediterranean Region; IMSEAR, Index Medicus for the South-East Asian Region; AIM, African Index Medicus.

#### Eligibility criteria

We searched for experimental and non-experimental studies that examined the effectiveness or that performed economic evaluations of the implementation of a behavioural health integration model on the management of depression and unhealthy alcohol use in primary healthcare in low- and middle-income countries. Articles eligible for inclusion were required to meet the following criteria: (a) studies included patients aged ≥18 years, of any gender and with a diagnosis of alcohol use disorder and/or depression of any severity; (b) studies performed with a population living in low- and middle-income countries as per the World Bank country income classification^28^ during the year that the study started; (c) studies included patients who received mental health services (in depression and/or alcohol use disorder) in fully or partially integrated primary health services in low- and middle-income countries^29^ and (d) studies included the integration of pharmacological or psychological interventions, or a combination of both. All study designs were considered. We excluded single-case studies, presentations, abstracts, notes, corrections and studies that did not report effectiveness or economic evaluation outcomes.

### Phases 2 and 3: abstract and full-text review

**Fig. 1:**
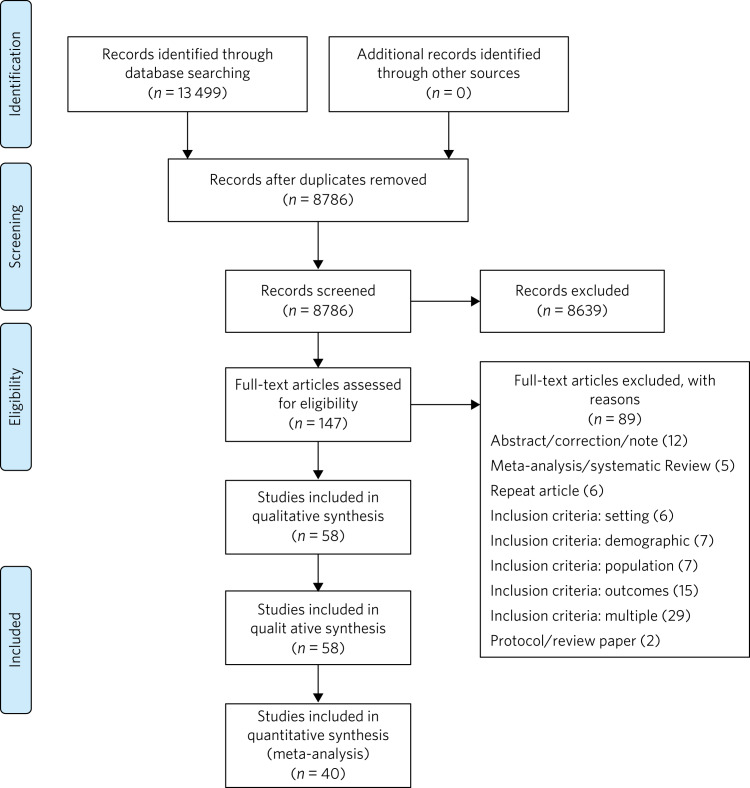
Flow chart of search results.

Using previously agreed inclusion criteria, three teams of two researchers per team each independently screened a third of the abstracts and titles (approximately 2918 abstracts). Disputed references (around 11%) were sent to an arbiter who settled the discrepancies. A total of 147 articles (roughly 1.7% of original abstracts) were selected for full-text appraisal of inclusion criteria. In each team in phase 3, one reviewer acted as the primary reviewer, the second reviewer evaluated the work for discrepancies and an arbiter settled the differences. This full-text review identified 58 articles meeting inclusion criteria, 40 of which met study design criteria and provided sufficient statistical data to be included in a subsequent meta-analysis (Fig. 1).

### Phase 4: data extraction

In phase 4, we completed an in-depth data extraction with a previously designed form (Table 2). We also completed a standardised assessment of bias of all the included randomised controlled trials, using methods described in the Cochrane Collaboration's tool for assessing risk of bias.^30^ This assessment of bias included a team of two of the authors independently evaluating the studies across seven categories: random sequence generation, allocation concealment, blinding of participants and personnel, blinding of outcome assessment, incomplete outcome data, selective reporting and other bias. Studies were rated across these categories as having a ‘low risk of bias’, ‘unclear risk of bias’ or ‘high risk of bias’, and all discrepancies on risk classifications were resolved by a third author.

**Table 2:** Phase 5: list of variables

Setting of care
Where does the screening take place?
Where does the intervention occur?
Where does the follow-up take place?
Human resources
Who screens?
Who delivers the clinical intervention?
Who prescribes?
Who provides additional services?
How is supervision done?
Who refers the patient?
Who receives the referral?
Who pays the salaries of the intervention team?
How is the intervention team paid?
Who provides training?
Who receives training?
Interventions
Description of the intervention
Description of the training
What is the relation between the clinical intervention team and the PHC?
Elements of the collaborative care management model
Presence of care managers
Role of care managers in symptom assessment
Role of care managers in monitoring treatment adherence
Composition of multidisciplinary teams
Existence of patient education programmes
What is the role of patient data in the care of the patient?

PHC, primary health center.

### Phase 5: patterns in the organisation of care of behavioural health integration models

During phase 4, we noticed patterns in the reorganisation of care that enabled the delivery of integrated mental health interventions in the treatment arms. We used the 2018 Joint Commission Ambulatory Accreditation Quality of Care Standards to assess the dimensions of quality of care involved in these reorganisations of care.^31^ We updated the data extraction form used in phase 4, adding variables related to organisation of care, and reviewed all articles once again (Table 2). We extracted data by structural coding. During phases 4 and 5, one researcher (L.C.) extracted these data, with a second reviewer (S.B.) assessing the data for discrepancies. An arbiter resolved any differences that the researchers found.

## Results

### Description of the included studies

The 58 included publications corresponded to 53 unique studies assessing the effectiveness or performing an economic evaluation of behavioural health integration in 19 low- and middle-income countries. Of the 58 articles, 20 took place in a rural setting, 3 took place in semi-urban settings, 23 took place in urban settings, 7 took place in both rural and urban settings, and the settings of the remaining 5 are unclear or unable to be categorised. The vast majority of the studies introduced interventions in primary healthcare, although some interventions were introduced in communities, over the phone or in public hospitals. Of the 55 articles for which this categorisation applies, 22 articles studied only women, 3 studied only men and 30 studied both women and men. Eleven articles came from low-income countries, 19 articles came from lower-middle income countries and 28 articles came from upper-middle income countries. Based on the WHO regional grouping classification,^32^ 22 articles came from Africa, 15 articles came from the Americas, 13 articles came from Southeast Asia, 8 articles came from the Eastern Mediterranean region and 2 articles came from the Western Pacific region. Two studies counted for both India and Pakistan, which are classified in two different WHO regions (Supplementary Table 1).

We found that of the 58 total articles, 46 focused on depression, 9 focused on unhealthy alcohol use and 3 focused on both illnesses. Further, 49 assessed the effectiveness of integration models, 6 performed economic evaluations, 2 performed both assessments and 1 presented a descriptive analysis. Of the 51 publications reporting effectiveness data (8 reported economic evaluation data), 40 focused on depression, 9 focused on alcohol use and 2 presented data related to both conditions. These 51 publications also varied in study design: 36 studies were randomised controlled trials, 7 were quasi-experimental studies and 8 were observational studies.

**Fig. 2:**
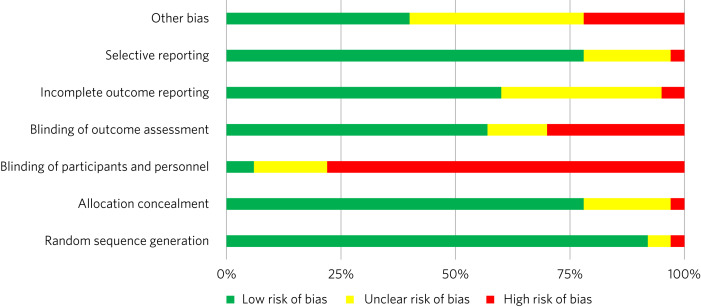
Consolidated risk of bias graph.

We found a high risk of selection bias among our studies, owing to a lack of blinding of ‘participants and personnel’; more than 75% of our studies had a ‘high risk’ of this type of bias. This finding was not surpirising given the nature of the mental health interventions, for many of which it was not possible to blind the study participants. The effect on the evidence quality is low since the authors used standardised and objective methods for outcome measurement (Fig. 2).

### Results of the effectiveness studies

#### Depression

Forty-two articles presented effectiveness data on depression care (Supplementary Table 1). The most frequently studied primary outcome was severity of depression. The treatment arm usually received a psychological intervention like cognitive–behavioural therapy, problem-solving therapy, psychoeducation or interpersonal therapy, whereas the care for the control arm was enhanced with screening only. Thirty-six articles reported that the integration groups had a greater reduction in depression severity than the control groups. Of these 36 articles, 5 articles reported effect size. Overall, differences between arms sustained through secondary follow-ups ranged from 3 months to 36 months post-intervention. Three of the articles that found no difference between the control and treatment groups offered only a general training on mental healthcare to their lay or primary healthcare workers expecting to change their clinical behaviours.^33–35^ Two other publications lacking positive findings selected primary outcomes highly susceptible to confounding.^36,37^ In India, Pradeep et al^38^ sought to improve treatment-seeking behaviours and adherence to antidepressant medications by enhancing usual care. In Iran, Malakouti et al^39^ sought to reduce the number of suicides. In Pakistan, Husain et al^40^ compared the effectiveness of psychotherapy to antidepressant medications in reducing depression and improving quality of life. This group compared two integrated interventions without contrasting it to usual care and found no difference between these two arms.

#### Alcohol use

Eleven articles reported effectiveness data related to unhealthy alcohol use.^41–51^ All 11 used a reduction in alcohol consumption as their primary outcome. Ten of these articles were randomised controlled trials (Supplementary Table 1). Of these, five favoured the intervention arm,^41,42,45,48,50^ five did not show differences between arms or after the intervention^43,44,46,49,51^ and one had unclear results.^47^ For the studies that favoured the intervention arm, only one paper reported effect size (*d* = 0.95).^52^ For most studies, secondary follow-ups showed that statistically significant differences sustained over 3 months to 12 months post-intervention. The two most commonly delivered interventions were screening followed by brief intervention or by motivational interviewing. In Thailand, Noknoy et al,^41^ and in South Africa, Rendall-Mkose et al,^48^ found that motivational interviewing arms produced better outcomes than enhanced usual care in improving patient outcomes. In Kenya, L'Engle et al^45^ found that screening and a brief intervention can reduce self-reported alcohol consumption among female sex workers at risk for HIV. In South Africa, Mertens et al^44^ found that patients receiving brief intervention had significantly reduced Alcohol, Smoking and Substance Involvement Screening Test scores. However, in South Africa, Peltzer et al,^46^ and in Thailand, Assanangkornchai et al,^51^ found no difference between brief intervention and simple advice as both arms similarly reduced alcohol consumption. Similarly, in South Africa, Sorsdahl and Petersen,^49^ in an uncontrolled before-and-after study, did not find a significant decrease in alcohol use in pregnant women following a brief intervention.

In Kenya, Papas et al^50^ found effectiveness of cognitive–behavioural therapy over treatment as usual in reducing use and attaining abstinence in patients living with HIV. In India, Nadkarni et al^42^ developed a novel and culturally adapted psychotherapy for unhealthy alcohol use delivered by lay health workers, called counselling for alcohol problems. Patients receiving counselling for alcohol problems showed higher remission rates and higher proportion of alcohol-free days than individuals receiving enhanced usual care.

### Results of the economic evaluation studies

We identified eight studies performing economic evaluations (Supplementary Table 1). These studies were conducted in Chile, India, Mexico, Nigeria and Pakistan. Six of these studies assessed interventions related to depression,^52–57^ one study evaluated interventions related to alcohol use^42^ and one publication evaluated both conditions.^58^ Four of these studies used a healthcare perspective in their economic analysis,^52,56–58^ two used a societal perspective^54,55^ and two used both perspectives.^42,53^ In economic analysis, a healthcare perspective includes all costs and benefits directly affecting patients, providers and payers.^25^ Conversely, a societal perspective takes a broader approach to include healthcare plus other private and public benefits and costs related to a given condition. Concerning the type of economic analysis, five studies performed a cost-effectiveness analysis,^52,55–58^ two completed a cost–utility analysis^42,53^ and one study performed both types of economic analysis.^54^ Three studies used modelling techniques^52,57,58^ and five studies used experimental data from effectiveness trials.^42,53–56^

Two of the articles using modelling techniques^52,58^ utilised the sectoral approach to cost-effectiveness analysis developed by the WHO's ‘Choosing Interventions that are Cost-Effective’ initiative.^59^ These studies found that a combined intervention of medications and therapy plus proactive case management yielded the highest effectiveness among the alternative options. In a study in Nigeria, Gureje et al^58^ found that a combination of tricyclic antidepressants, psychotherapy and proactive case management had the highest cost-effectiveness ratio, closely followed by tricyclic antidepressants only (approximately 0.5% less cost-effective). In Mexico, Del Carmen et al^52^ found that a medication-only intervention was the most cost-effective, followed by a combination of medication, psychotherapy and proactive case management (approximately 10.5% less cost-effective).

From a healthcare perspective, the economic analyses of the experimental studies showed that the intervention arms had increased effectiveness and costs.^42,53,54,56^ The increased direct costs were associated with increased utilisation of healthcare personnel and medications. These interventions were deemed cost-effective based on the acceptability threshold and commonly accepted values for cost-effectiveness. Using a Markov model, in Chile, Siskind et al^57^ modeled the cost-effectiveness of a stepped-up care intervention throughout the lifetimes of a cohort of Chilean adult females. This study also evidenced increased direct healthcare costs, but found integration to be very cost-effective.

Notably, from a societal perspective, these interventions were found to reduce costs, usually associated with decreased time costs to patients and families, as well as fewer productivity losses.

### Models of behavioural health integration

The control arm of the experimental studies included in this review used treatment as usual (minimal or no services) or enhanced usual care. In contrast, to integrate mental healthcare, the treatment arms redesigned their care by selecting at least one of the seven strategic intervention options (Table 3). Each strategic intervention option represents a discrete active enhancement to the primary healthcare affecting workforce capacity, information management or daily flow of care.^31^ The behavioural health integration models tested in the research are made up of one or more strategic intervention options. Furthermore, the treatment arms of the 53 studies included in this secondary analysis clustered around 6 of these integration models (Table 4). We were unable to include five studies in this secondary analysis: two owing to paucity of data^55,60^ and three owing to use of modelling methods for economic evaluation that did not study any specific behavioural health integration model.^52,57,58^

**Table 3:** Organisational strategic options used in the integration models

Strategic intervention options	Description	Joint Commission standard of ambulatory quality of care (standard label)
1	General training on mental healthcare for lay and primary healthcare workers	Human resources (H.R. 01.05.03)
2	Specific training on mental healthcare skills and interventions for lay and primary healthcare workers	Human resources (H.R. 01.05.03)
3	Addition of dedicated lay or primary healthcare workers to provide mental health services	Human resources (H.R. 01.06.01)
4	Addition of specific mental healthcare tasks to existing lay or primary healthcare workers	Human resources (H.R. 01.05.03) Provision of care (P.C. 02.01.01)
5	Increased coordination between lay or primary healthcare workers with mental health workers	Provision of care (P.C. 02.01.05)
6	Strategic data management to improve patient outcomes	Provision of care (P.C. 02.01.05) Information management (I.M. 02.02.01)
7	Utilisation of care manager or care coordinator	Provision of care (P.C. 02.01.01) Provision of care (P.C. 02.01.05)

**Table 4:** Summary of the integration models and the organisational strategic options used in each model

Strategic intervention options								
	1	2	3	4	5	6	7	
Models of behavioural health integration	General training on mental healthcare for lay and primary healthcare workers	Specific training on mental healthcare skills and interventions for lay and primary healthcare workers	Addition of dedicated lay or primary healthcare workers to provide mental health services	Addition of specific mental healthcare tasks to existing lay or primary healthcare workers	Increased coordination between lay or primary healthcare workers with mental health workers	Strategic data management to improve patient outcomes	Utilisation of care manager or care coordinator	Type of healthcare workers involved in the model
1. General training on mental healthcare for lay health workers and primary health workers	Yes	No	No	No	No	No	No	LHW, PHW,
2. Mental healthcare interventions delivered by lay health workers	Yes	Yes	Not essential but could be added	Not essential but could be added	No	No	No	LHW
3. Mental healthcare interventions delivered by primary healthcare workers	Yes	Yes	Not essential but could be added	Not essential but could be added	No	No	No	PHW,
4. Consultation liaison	Not essential but could be added	No	No	Yes	Yes	No	No	LHW, PHW, MHW
5. Stepped care	Yes	Yes	Not essential but could be added	Yes	Yes	Yes	No	LHW, PHW, MHW
6. Collaborative care	Yes	Yes	Yes	Yes	Yes	Yes	Yes	LHW, PHW, MHW, care coordinator

Those strategic options deemed essential for each model are marked with a ‘Yes’ sign. LHW, lay health worker; PHW, primary health worker; MHW, mental health worker.

Models 1–3 rely on building the capacity of non-specialist health workers in primary care, and they represent different task-sharing models. These models heavily depend on organisational strategic intervention options 1–4. Unlike models 4–6, the first three models do not depend on increased coordination between primary health workers, or between the primary healthcare site and other healthcare organisations. There are also minimal modifications in the daily flow of care. Starting in model 4, these integration models increasingly require collaboration and information flows across multidisciplinary teams. Patient-level data is strategically used to improve the care of patients in models 5 and 6.

#### Model 1: general training on mental healthcare for lay health workers and primary health workers

Seven studies met the criteria for model 1. This model utilises strategic intervention option 1. In this model, following training only, lay or primary healthcare workers (general physicians and nurses) are expected to have an increased ability to diagnose and treat mental health conditions adequately. For example, a study in Colombia^61^ compared the diagnostic accuracy and effectiveness of general physicians who had received formal training on mental healthcare to that of similar general physicians who did not. The study found that patients in the intervention arm received better treatment, had increased rates of retention and had lower depression scores than the control arm.

#### Model 2: mental healthcare interventions delivered by lay health workers

Twenty studies met the criteria for model 2. In addition to general training (strategic intervention option 1), lay health workers also receive specific training, ranging from a few days to 2 weeks (strategic intervention option 2), that prepares them to deliver targeted interventions, such as screening, problem-solving or interpersonal therapy. This model requires that the primary healthcare site either hires new lay health workers (strategic intervention option 3) or reassigns those currently delivering other services (strategic intervention option 4). This model may benefit from having primary health workers (general physicians or nurses) perform supervisory functions. Using this model, a study in rural South Africa^62^ compared the effectiveness of a 12-week course of interpersonal therapy delivered by lay health workers to enhanced care in the reduction of depression among low-income women. Patients in the intervention arm showed a significant decrease in depression symptoms upon completion of the 12-week course.

#### Model 3: mental healthcare interventions delivered by primary health workers

Eleven studies met the criteria for model 3. This model uses strategic intervention options 1 and 2 plus either strategic intervention option 3 or 4. This model often uses flow of care modifications to carve out dedicated time for the primary health workers. A study in rural Thailand^41^ compared the effectiveness of nurse-delivered brief interventions versus treatment as usual (e.g. no brief intervention) in the reduction of alcohol consumption among low-income males. Patients in the intervention arm reported a more substantial decrease in drinks per drinking day and frequency of unhealthy drinking assessed 6 months after the intervention.

#### Model 4: consultant liaison

One study met the criteria for model 4. This model offers the primary health worker access to onsite or telemedicine consultation services from a mental health worker such as psychologists or psychiatrists (strategic intervention option 5), although the primary health worker continues to be the main provider. Consultation services include education, problem-solving and feedback to the primary health worker regarding diagnostic or treatment strategies.^63,64^ Strategic intervention option 5 is essential in this model. A study in Chile^36^ compared the effectiveness of treatment delivered by general physicians with access to online psychiatric consultation services with that of those without access to this support in the management of urban women diagnosed with depression. Patients in the intervention arm had a statistically significant reduction in their depression scores compared with those in the control arm at 3 months of the intervention.

#### Model 5: stepped care

Eight studies met the criteria for model 5. This model provides a structured way to match treatment intensity with the patient's needs.^65^ More complicated patients are cared for by a mental health worker (strategic intervention option 5), whereas more straightforward cases remain under the care of the primary health worker (strategic intervention option 4). Some studies used lay health workers, creating a three-level stepped care model (strategic intervention option 3). This model distinctively uses a set of clinical criteria and a pathway of care to systematically step up or step down each case. Thus, this model adds outcome tracking to inform the level of care provided to a patient (strategic intervention option 6). A post-rollout evaluation in Iran^39^ assessed the effectiveness of a suicide prevention strategy for adults with depression. In this programme, a lay health worker reached out to patients to screen them for depression and referred positive cases to a primary health worker for management and stabilisation. In turn, the primary health worker referred refractory cases to psychiatrists who delivered specialised services.

#### Model 6: collaborative care management

Six studies met the criteria for model 6. There is variation in the literature regarding the components of collaborative care management, and there are different levels of complexity within collaborative care management itself.^66^ For this systematic review, strategic intervention options 6 and 7 are considered critical. Other collaborative care management elements, such as linkage to community resources, patient self-management support, regular case consultation from a psychiatrist, provider decision support and healthcare organisation support, could also be present.

In China, a study^67^ compared the effectiveness of two modalities of depression treatment for adults aged ≥60 years. In the intervention arm, general physicians (strategic intervention option 4), primary care nurses serving as care managers (strategic intervention option 7), and psychiatrists (strategic intervention options 5) comprised the treatment team. General physicians received written guidelines for the treatment of depression, as well as in-depth training in the prescription of antidepressants and the appropriate use of referrals to the psychiatrist (strategic intervention options 1–3). Nurses acting as care managers provided psychoeducation to patients and families, assistance with communication between patients and providers, and support for the patient's adherence to treatment. A study psychiatrist was made available in case of referrals. General physicians in the control arm only received written guidelines for depression treatment, patients’ PHQ-9 scores and major depression diagnoses from the screening stage. Patients in the intervention arm experienced significantly greater reductions in Hamilton Rating Scale for Depression scores than those in the control arm.

## Discussion

### Overview

The findings of this systematic review support the effectiveness of different models of integrating depression and unhealthy alcohol use care in primary healthcare in low- and middle-income countries. Patients receiving treatment in the integrated models tend to have better outcomes compared with those receiving regular care. The evidence appears more robust for depression than for unhealthy alcohol use. The economic analyses indicate that integrated models have higher direct costs to primary health, and that from the healthcare perspective, these models are cost-effective. It is also possible that behavioural health integration saves costs to society by increasing productivity and decreasing time losses, among other benefits. The typology proposed in this article can improve the understanding of the different models of behavioural health integration in low- and middle-income countries. This information can be valuable for policy makers and hospital managers responsible for the organisation and delivery of care. Additional implementation studies are required to further characterise the different models of integration and to understand better the conditions needed for the implementation of each of them.

### Increased effectiveness across different settings and populations

The studies included in this review showed that integrated models can improve patient outcomes in different subtypes of depression such as perinatal depression, late-in-life depression, comorbid depression and HIV, and depression associated with trauma disorders in war-affected areas.^68,69^ Previous research shows that some psychological treatments can be as effective as antidepressant medications, with higher retention rates and better continuing outcomes.^70,71^ We found that different psychotherapies can be effectively delivered by an array of integration models. These can be more culturally adaptable,^72^ and possibly less stigmatising than medication-based treatments. They can also be potentially scalable in low- and middle-income countries contexts where community bonding is strong, labour is more available and procurement and distribution chains for pharmaceuticals are precarious.

This systematic review suggests that integration of care for unhealthy alcohol use might produce better outcomes for the general population, pregnant women and people living with HIV in low- and middle-income countries. The control arm of seven included trials compared screening and minimal psychoeducation to screening and brief intervention or motivational interviewing offered in the intervention arms.^41–44,46,48,51^ The enhancement of the control arms could account for the non-positive results, particularly in settings where neither screening nor minimal psychoeducation is routinely offered in primary healthcare. There is evidence that screening alone can affect the patients’ behaviours, which could explain the lack of difference between arms in some studies.^73^ Kaner et al^15^ found that screening and brief interventions can reduce alcohol consumption in hazardous and harmful drinkers compared with minimal or no interventions in primary healthcare in high-income countries. Although the findings of the articles included in this review are similar to those in high-income countries, we found few studies targeting unhealthy alcohol use that fulfilled our selection criteria, which may affect the generalisability of our findings. More research in the adequate care of unhealthy alcohol use in low- and middle-income countries is needed.

### Increased funding is a necessary, but not sufficient condition to increase access to care

The economic evaluations included in this review indicate that integrated models may result in increased direct costs to primary health, stemming from increased utilisation of personnel and medications. Nevertheless, they may save costs to society.^42,53,57^ These findings are similar to those found in high-income countries.^74^ Given the low levels of spending on mental healthcare in many low- and middle-income countries,^11^ where the vast majority of primary healthcare sites do not provide access to mental health services, the finding that increasing the availability of mental health services increases direct costs should not surprise. Since low- and middle-income countries favour funding of mental health hospitals,^11^ new resources should be earmarked to sustain behavioural health integration in primary care. Moreover, the way in which the health system pays or transfers funds to primary healthcare should also be carefully examined. Health economics literature has extensively shown that these payment mechanisms are key determinants of providers’ behaviours.^75^ The most commonly used payment mechanisms in many low- and middle-income countries are out-of-pocket, capitation and historically determined allocations;^76^ however, since they are not explicitly linked to outputs or outcomes, they do not provide adequate incentives to increase the availability of integrated services. Recent research in high-income countries has studied the development of new payment mechanisms to promote increased integration and coordination of care for populations with multiple chronic comorbidities.^77,78^ Additional research is needed to specifically adapt payment mechanisms to offset the increased direct costs related to behavioural health integration, thus encouraging primary care in low- and middle-income countries to increase the availability of services.^75^ Importantly, public and private providers may respond differently to these incentives, as evidenced in several studies included in this review where integration models affected patient outcomes in public, but not in private organisations.^79,80^

### Typology of integration for low- and middle-income countries: a tool for decision makers

The reviewed studies tested a variety of models of integrated care for depression and unhealthy alcohol use. We offer a typology of the models in Table 4 and show how they are built from one or more of seven organisational strategic intervention options. The typology aims to assist decision makers in selecting the models that are likely to work over time in their setting. The strategic intervention options, and the models that flow from them, are not hierarchical but do vary in terms of cost, complexity and how much organisational capacity they require to implement and sustain. Decision makers can choose models that match the characteristics and capacity of their health system and primary healthcare. An appealing complex model may not be the right choice if it is too expensive or requires too much change from the workforce to be implemented or sustained.^81^ An integration model that fits well with current programmes and available resources might have a greater effect over time. Since complex strategic intervention options require more resources to implement and sustain, they are more likely to be chosen in higher income nations. Decision makers must consider effectiveness, acceptability, sustainability and scalability in choosing a model to meet their system's needs.

### Limitations

This review has several limitations. Some of the studies included in this review were not rigorously designed trials and did not have adequate comparison conditions. For example, some of the studies were post-rollout evaluations and other were pragmatic or quasi-experimental trials. This review focused on the care of depression and alcohol use disorder. Therefore, our findings may not be generalisable to other mental or substance use disorders. Similarly, we excluded studies assessing the effectiveness of psychological interventions for these two conditions in low- and middle-income countries when they did not reflect the integration of these treatments into existing primary healthcare settings. Nonetheless, this systematic review offers important insights into the value and implementation of integrated models in global mental healthcare.

### Implications for the global mental health policy field

The findings of this review build on a wealth of knowledge strongly supporting the value of integrating mental healthcare into primary care.^77,78^ The next generation of research should aim to understand the arrangements at the system and organisational levels necessary to scale up integrated models in low- and middle-income countries and to promote the delivery of quality healthcare. In particular, we need to strengthen the instruments used to measure the quality of integration in low- and middle-income countries. Similarly, understanding the reasons underpinning the rampant mental health workforce shortage is critical because behavioural health integration heavily relies on existing and newly available workforce. To a certain degree, a combination of additional funds and targeted payment mechanisms can provide the right incentives to overcome some of these implementation challenges and to sustain quality of mental healthcare. Further research related to payment mechanisms in primary care in low- and middle-income countries is therefore critically needed.

The global mental health field can learn from other successful global health movements. Efforts to address HIV, reduce child mortality and improve maternal health were able to permeate political spaces and become global health priorities, channelling substantive resources, some of which have been used to integrate these services into primary care. At the national level, the experiences of Chile and Zimbabwe where research studies influenced the governments to expand publicly funded mental healthcare programmes can illustrate processes that occupied the political agenda and affected public policy.^82,83^ Furthering our understanding of the operation of behavioural health integration into primary care and bettering our ability to scale up these integrated models can help close the treatment gap and raise the quality of mental care in low- and middle-income settings.

## About the authors

**Leonardo Cubillos** (MD, MPH) is a senior policy advisor for the Center for Technology and Behavioral Health at Geisel School of Medicine at Dartmouth College, USA. **Sophia M. Bartels** (BA) is a research assistant at the Center for Technology and Behavioral Health at Geisel School of Medicine at Dartmouth College, USA. **William C. Torrey** (MD) is Professor and Vice Chair for Clinical Services for the Department of Psychiatry at Geisel School of Medicine at Dartmouth, USA. **John Naslund** (PhD) is a research fellow in the Department of Global Health and Social Medicine at Harvard Medical School, USA. **José Miguel Uribe-Restrepo** (MD, MPH) is Chairman of the Department of Psychiatry at Pontificia Universidad Javeriana, Colombia. **Chelsea Gaviola** (MPH, BA) is a medical student at Geisel School of Medicine at Dartmouth College, USA. **Sergio Castro Díaz** (MD) is a researcher in the Department of Clinical Epidemiology and Biostatistics at Pontificia Universidad Javeriana, Colombia. **Deepak T. John** (BA) is a research coordinator at the Center for Technology and Behavioral Health at Geisel School of Medicine at Dartmouth College, USA. **Makeda J. Williams** (PhD, MPH) is Chief of the Center for Global Mental Health Research at the National Institute of Mental Health, USA. **Magda Cepeda** (MD, PhD) is a research coordinator in the Department of Clinical Epidemiology and Biostatistics at Pontificia Universidad Javeriana, Colombia. **Carlos Gómez-Restrepo** (MD, MSc) is Dean of the Faculty of Medicine at Pontificia Universidad Javeriana, and Professor in the Departments of Clinical Epidemiology and Biostatistics, and Psychiatry and Mental Health at Pontificia Universidad Javeriana, Colombia. **Lisa A. Marsch** (PhD) is Director of the Center for Technology and Behavioral Health at Geisel School of Medicine at Dartmouth College, USA.

## Supplementary material

For supplementary material accompanying this paper visit https://doi.org/10.1192/bjb.2020.35. click here to view supplementary material
