# Supplementary material for: The effectiveness and cost-effectiveness of integrating mental health services in primary care in low- and middle-income countries: systematic review
Source: BJPsych Bull. 2021 Feb;45(1):40–52. doi: 10.1192/bjb.2020.35 (PMC8058938; doi:10.1192/bjb.2020.35)
Supplement: Supplementary file 1 [file S2056469420000352sup.zip › S2056469420000352sup003.docx]

| **Author (Year)** | **Country (Income Classification)****** | **Study Design** | ***Evaluation* & Condition** | **Intervention (if applicable) &**  **Outcomes** | **BHI Model** |
| --- | --- | --- | --- | --- | --- |
| Al-Faris et al., (1997)(1) | Saudi Arabia** (UMIC) | RCT | *Effectiveness*  Depression | Training of GPs to Diagnose Depression  --Diagnostic accuracy improved most in intervention group, minimally in control group 1, and worsened in control group 2 | Model 1 |
| Sabir Ali et al., (2003)(2) | Pakistan (LIC) | RCT | *Effectiveness*  Depression in women | LHW-Delivered PST, CBT and Supportive Therapy  --Depression scores reduced significantly in the LHW intervention group vs. control | Model 2 |
| Ali et al., (2010)(3) | Pakistan (LMIC) | Controlled Before-and-After Study | *Effectiveness*  Depression – Postnatal depression | LHW-Delivered Counseling  --Depression reduced significantly in intervention and control groups  --Intervention group fared better in recovery, recurrence, and time before relapse | Model 2 |
| Alvarado and Rojas* (2011)(4) | Chile (UMIC)*** | Observational Study | *Effectiveness*  Depression | Multi-Faceted Stepped Care Program  --Severity of depression was underestimated by primary care teams  --Patients with mild, moderate, and severe depression had BDI reductions of 35%, 37%, and 13%, respectively | Model 5 |
| * Alvarado et al., (2005)(5) | Chile (UMIC) *** | Observational Study | *Effectiveness*  Depression in women | Stepped Care Program  --Depression symptom severity reduced significantly  --Symptoms reduced most in those who had the most severe symptoms at baseline | Model 5 |
| *Antini and Alvarado (2008)(6) | Chile (UMIC) *** | Observational Study (Cohort) | *Effectiveness*  Depression in women | Stepped Care Program  --Depression and quality of life scores improved significantly in those who remained in treatment vs. those who abandoned treatment | Model 5 |
| * Araya et al., (2003)(7) | Chile (UMIC)*** | RCT | *Effectiveness*  Depression in women | Multi-Faceted Stepped Care Intervention  --HAMD scores decreased significantly in intervention vs. controls and at 6-month follow-up | Model 5 |
| * Araya et al., (2006)(8) | Chile (UMIC)*** | Economic analysis of an RCT | *Economic Evaluation / Cost-Effectiveness*  Depression in women | Multi-Faceted Stepped Care Intervention  --Intervention group had 50 more depression-free days over 6 months vs. controls  --Intervention was marginally more expensive than control | Model 5 |
| Assanangkornchai et al., (2015)(9) | Thailand (UMIC) | RCT | *Effectiveness*  Problematic Alcohol Use | Nurse-Delivered ASSIST followed by BI or SA  --Both groups reduced alcohol consumption; there was no significant difference between them | Model 3 |
| Baker-Henningham et al., (2005)(10) | Jamaica (LMIC) | RCT | *Effectiveness*  Depression – Perinatal depression | LHW-Delivered Home Visits/Childhood Stimulation  --Intervention mothers reported a significant reduction in freq. of depressive symptoms vs. controls----Mothers receiving more visits benefitted most | Model 2 |
| * Berbesi et al., (2010)(11) | Colombia (LMIC) | Observational Study | *Effectiveness*  Depression | Training of PHW  --Compared to control patients, intervention patients experienced a greater “success rate” that includes the probability of receiving adequate treatment (as determined by a psychiatrist), completing the course of treatment, and not presenting depression 3 months post-intervention | Model 1 |
| Buttorff et al., (2012)(12) | India (LMIC) | Economic analysis of an RCT | *Economic Evaluation / Cost-Utility AND Cost-Effectiveness*  Depression | Multidisciplinary Team-Delivered Intervention  -- More costly and more effective from the health system perspective  -- Less costly and more effective from the societal perspective  --Probability close to 1 that this intervention is cost-effective | Model 6 |
| Chen et al., (2015)(13) | China (UMIC) | RCT | *Effectiveness*  Depression | CCM  --HAMD scores reduced significantly in CCM vs. EUC | Model 6 |
| * Chibanda et al., (2011)(14) | Zimbabwe (LIC) | Uncontrolled Before-and-After Study | *Effectiveness*  Depression | LHW-Delivered Psychotherapy  --SSQ scores fell proportionally to number of sessions attended | Model 2 |
| Chibanda et al., (2014)(15) | Zimbabwe (LIC) | RCT | *Effectiveness*  Depression - Postnatal depression | Peer Counselor-Delivered Group PST  --Intervention group’s depression symptoms were reduced significantly more than amitriptyline group | Model 2 |
| * Chibanda et al., (2016)(16) | Zimbabwe (LIC) | RCT | *Effectiveness*  Depression | LHW-Delivered Psychotherapy  --Intervention group had significantly fewer SSQ symptoms and risk of depression symptoms than controls at 6 months | Model 2 |
| Chisholm et al., (2000)(17) | India/  Pakistan (LIC/LIC) | Uncontrolled Before-and-After Study | *Economic Evaluation / Cost-Effectiveness*  Depression | Cost/Outcome Comparison of Integrated and Standard PHCs  --In 3 of 4 localities, symptoms, disability, and quality of life improved  --Economic costs reduced | Unclear |
| Chowdhary et al., (2016)(18) | India (LMIC) | Pilot RCT | *Effectiveness*  Depression | Lay Counselor-Delivered Psychotherapy  --Depression prevalence reduced in intervention arm vs. control arm after 2 months | Model 2 |
| Del Carmen Lara-Munoz et al., (2010)(19) | Mexico (UMIC) | Modelling using WHO-CHOICE sectoral approach | *Economic Evaluation / Cost-Effectiveness*  Depression | Identifying the Most Cost-Effective Interventions  --Single treatment: SSRI  --Combination: Proactive case management, brief psychotherapy, and antidepressants | Modelling methods for economic evaluation |
| Fairall et al., (2016)(20) | South Africa (UMIC) | RCT | *Effectiveness*  Depression | Nurse Training to Diagnose & Treat Mental Disorders  --Treatment intensification rates and case detection of depression did not differ between arms | Model 1 |
| Fritsch et al., (2007)(21) | Chile (UMIC)*** | RCT | *Effectiveness*  Depression in women | Pharmacotherapy with Telephone Monitoring  --Significant improvement in HAMD & SF-36 scores in intervention group vs. controls at 3 months and 6 months | Model 3 |
| Garcia-Pena et al., (2015)(22) | Mexico (UMIC) | RCT | *Effectiveness*  Depression in the elderly | Nurse-Led CBT  -- PHQ9 scores decreased by ≥5 points more in intervention patients than control patients | Model 3 |
| Gómez-Restrepo et al., (2007)(23) | Colombia (LMIC) | Uncontrolled Before-and-After Study | *Effectiveness*  Depression | Training of GPs to Diagnose Depression  --There was an increase in depression diagnosis in patients at moderate or high risk for depression | Model 1 |
| Gureje et al., (2007)(24) | Nigeria (LIC) | Modelling using WHO-CHOICE sectoral approach | *Economic evaluation/Cost-Effectiveness*  Depression AND Problematic alcohol use | Identifying the Most Cost-Effective Interventions  --Depression: Combination of TCA, psychotherapy, and case management  --Alcohol use: Random breath testing for motor vehicle drivers | Modelling methods for economic evaluation |
| Husain et al., (2014)(25) | Pakistan (LMIC) | RCT | *Effectiveness*  Depression in women | LHW + Psychologist-Delivered CBT  --No significant difference in depression reduction and quality of life between the group CBT and antidepressant (control) arms | Model 2 |
| James et al., (2002)(26) | India/Pakistan (LIC/LIC) | Observational Study | *Neither Effectiveness nor Economic Evaluation*  Depression | Identifying the Most Influential Factors in Service Utilization Patterns  -- Cost, distance from treatment center, perception of ineffective care, and stigma concerns | Unclear |
| Jenkins et al., (2013)(27) | Kenya (LIC) | RCT | *Effectiveness*  Depression | Training of PHWs  --Did not improve diagnostic rate of mental disorders  --Improved patient outcomes in routine clinical practice | Model 1 |
| Kauye et al., (2014)(28) | Malawi (LIC) | RCT | *Effectiveness*  Depression | Training of PHC Staff to Diagnose Depression  --Significantly greater depression diagnosis rate in intervention vs. control | Model 1 |
| L’Engle et al., (2014)(29) | Kenya (LMIC) | RCT | *Effectiveness*  Problematic  alcohol use | Nurse Counselor-Delivered Brief Intervention  --Significantly greater reduction in alcohol use and binge drinking in intervention vs. control at 6 and 12 months. | Model 3 |
| Lima and Fleck (2011)(30) | Brazil (UMIC) | Observational Study (Cohort) | *Effectiveness*  Depression | No Intervention (Patients Receiving Standard Depression Treatment Studied Over Time)  --At 9 months, 42% still had major depression, 25% experienced remission, 9% treated with antidepressants | Model 1 |
| Malakouti et al., (2015)(31) | Iran (UMIC) | Uncontrolled Before-and-After Study | *Effectiveness*  Depression | Stepped Care Program  --After 1 year, suicide rate increased in one site and decreased in another site | Model 5 |
| Maulik et al., (2017)(32) | India (LMIC) | Observational Study | *Effectiveness*  Depression | LHW-Delivered Psychotherapy  --Symptoms reduced significantly between start and end of intervention in depressed patients | Model 2 |
| Mertens et al., (2014)(33) | South Africa (UMIC) | RCT | *Effectiveness*  Problematic  Alcohol Use | Nurse-Practitioner-Delivered Brief Motivational Intervention  --Rates of at-risk alcohol use did not differ between arms  -- Brief intervention patients had significant reduced ASSIST scores | Model 3 |
| Nadkarni et al., (2017)(34) | India (LMIC) | RCT | *Effectiveness AND*  *Economic Evaluation / Cost Utility*  Problematic Alcohol Use | Lay Counselor-Delivered Psychotherapy  -- Combined intervention and EUC reduced drinking more than EUC alone  --Incremental cost per additional remission was $217 with an 85% chance of being cost-effective | Model 2 |
| Nakimuli-Mpungu et al., (2015)(35) | Uganda (LIC) | RCT | *Effectiveness*  Depression in patients with HIV | Group Support Psychotherapy vs. Group Education  --At 3 months, no diff between psychotherapy and education arms  --At 6 months, psychotherapy arm had lower depression scores and higher function scores than education arm | Model 3 |
| Niemi et al., (2016)(36) | Vietnam (LMIC) | RCT | *Effectiveness*  Depression | Nurse and GP-delivered Psychoeducation & Yoga  --Intervention group had significantly greater reduction in PHQ9 scores vs. control | Model 3 |
| Noknoy et al., (2010)(37) | Thailand (UMIC) | RCT | *Effectiveness*  Problematic Alcohol Use | Nurse-Delivered Motivational Enhancement Therapy  --Self-reported drinking reduced in MET vs. control at 6 weeks, but no difference at 3 and 6 months | Model 3 |
| Oladeji et al., (2015)(38) | Nigeria (LMIC) | RCT | *Effectiveness*  Depression | Stepped Care Program  --Severity of depression symptoms reduced more in intervention than usual care groups | Model 5 |
| Papas et al., (2011)(39) | Kenya (LIC) | RCT | *Effectiveness*  Problematic alcohol use in patients with HIV | LHW-Delivered Psychotherapy  --Alcohol use reduced significantly from baseline in CBT patients, and alcohol abstinence was greater in CBT vs. controls | Model 2 |
| Patel et al., (2010)(40) | India (LMIC) | RCT | *Effectiveness*  Depression | CCM  --Intervention patients were more likely to recover from CMD than controls at 6 months | Model 6 |
| Patel et al., (2011)(41) | India (LMIC) | RCT | *Effectiveness*  Depression | CCM  --Decrease in prevalence of mental disorders, suicide attempts, days out of work and psychological morbidity in intervention vs. control groups in public facilities | Model 6 |
| Patel et al., (2017)(42) | India (LMIC) | RCT | *Effectiveness AND*  *Economic Evaluation / Cost Utility*  Depression (Moderate to Severe) | Lay Counselor-Delivered Psychotherapy  --HAP and EUC was more effective for moderate/severe depression than EUC alone in PHCs  -- Incremental cost per QALY of $9,333 with 87% chance of being cost-effective | Model 2 |
| Peltzer et al., (2013)(43) | South Africa (UMIC) | RCT | *Effectiveness*  Problematic alcohol use | Lay Counselor-Delivered Screening and BI  --Intervention and control (psychoeducation) reduced AUDIT scores for all groups  --No statistically significant effects | Model 2 |
| Petersen et al., (2012)(44) | South Africa (UMIC) | Quasi-Experimental | *Effectiveness*  Depression | Lay Health Worker-Delivered Group-Based IPT  --Intervention arm had significant reduction in symptoms vs. control arm at 12 and 24 weeks | Model 2 |
| Petersen et al., (2014)(45) | South Africa (UMIC) | RCT | *Effectiveness*  Depression in patients with HIV | Lay HIV-Counselor-Delivered Group-Based IPT  --PHQ9 scores improved significantly more in intervention than control patients | Model 2 |
| Pradeep et al., (2014)(46) | India (LMIC) | RCT | *Effectiveness*  Depression in women | CCM  --No significant difference in severity or quality of life in intervention vs. EUC  --Intervention group stayed on treatment for longer duration than EUC controls | Model 6 |
| Rahman et al., (2008)(47) | Pakistan (LMIC) | RCT | *Effectiveness*  Depression – Perinatal depression | LHW-Delivered CBT-Based Intervention  --At 6-month follow up, 23% of intervention group and 53% of controls met MDD criteria, difference maintained at 12 months | Model 2 |
| Rahman et al., (2016)(48) | Pakistan (LMIC) | RCT | *Effectiveness*  Depression in conflict zones | LHW-Delivered Psychotherapy  --At 3 months, intervention group had significantly lower HADS scores vs. controls | Model 2 |
| Ramarumo et al., (2016)(49) | South Africa (UMIC) | RCT | *Effectiveness*  Problematic Alcohol Use | Screening and Either BI or Educational Leaflet  -- No difference in alcohol consumption reduction between control and intervention arms | Model 3 |
| Rendall-Mkosi et al., (2013)(50) | South Africa (UMIC) | RCT | *Effectiveness*  Problematic  alcohol use in pregnant women | LHW-Delivered MI  --MI intervention group was less at risk for AEP vs. controls at 3 month and 12 month follow-up | Model 2 |
| Rojas et al., (2007)(51) | Chile (UMIC)*** | RCT | *Effectiveness* Depression in women | Multi-Faceted Stepped Care Intervention  --EPDS scores were lower for intervention vs. controls at 3 months and 6 months  --Fewer women took antidepressants at 3 months in intervention vs. control | Model 5 |
| Rojas et al., (2014)(52) | Chile (UMIC)*** | Quasi-Experimental | *Effectiveness*  Depression | GP Access to Online Consultation Services and Patient Access to Telephone Monitoring Service  --BDI scores decreased significantly more in intervention vs. controls | Model 4 |
| Rotheram-Borus et al., (2015)(53) | South Africa (UMIC) | RCT | *Effectiveness*  Depression AND  Problematic  alcohol use | Lay Health Worker Training/Home Visits  --Intervention mothers were less likely to report depressive symptoms than control mothers  --Better quality of life in intervention vs. controls at 36 months | Model 2 |
| Shidhaye et al., (2017)(54) | India (LMIC) | Observational Study | *Effectiveness*  Depression in rural settings | Multidisciplinary Community-Based Mental Health Program  --Contact coverage was 6x greater after 18 months, and mental health literacy improved | Model 6 |
| Siskind et al., (2010)(55) | Chile (UMIC)*** | Markov modelling using RCT data | *Economic Evaluation / Cost-Effectiveness*  Depression | Multi-Faceted Stepped Care Intervention  --Intervention ICER = 468/QALY vs. no treatment. Usual care ICER = $113/QALY vs. no treatment  --Very cost-effective given local GDP | Modelling methods for economic evaluation |
| Sorsdahl et al., (2015)(56) | South Africa (UMIC) | Uncontrolled Before-and-After study | *Effectiveness*  Depression AND  Problematic  alcohol use in pregnant women | Nurse and Counselor-Delivered Screening and Psychotherapy  --At 3-month follow up, depression scores and self-reported tobacco use decreased significantly, but no change in alcohol and other drug use | Model 3 |
| Tomlinson et al. (2016)(57) | South Africa (UMIC) | RCT | *Effectiveness*  Depression – Perinatal depression | LHW-Delivered Home Visits  --At 36 months, intervention mothers were less depressed than controls. Positive outcomes in offspring also found | Model 2 |
| Weiss et al., (2015)(58) | Iraq (UMIC) | RCT | *Effectiveness*  Depression among survivors of violence | LHW-Delivered Counseling (CETA) vs. Cognitive Processing Therapy  --CETA had large effect size for all outcomes, CPT had moderate effect size for trauma and depression | Model 3 |

**Table 3**

Description of included studies

* Publications associated with a public policy

** The World Bank Group reclassified Saudi Arabia as a HIC in 2004

*** The World Bank Group reclassified Chile as a HIC in 2012

**** Country level of income when study data was collected

Abbreviations:

Scales: PHQ9 = Patient Health Questionnaire, HAMD = Hamilton Depression Rating Scale, AUDIT = Alcohol Use Disorders Identification Test, SSQ = Shona Symptom Questionnaire, EPDS = Edinburgh Postnatal Depression Scale, HADS = Hospital Anxiety and Depression Scale, SF-36 = Short Form Health Survey

Staff: PHW = Primary Health Workers, LHW = Lay Health Workers, PHC = Primary Health Centers

Country level of income: LIC = Low-Income Country, LMIC = Lower Middle-Income Country, UMIC = Upper Middle-Income Country, HIC = High-Income Country

Study design: RCT = Randomized Controlled Trial, EUC = Enhanced Usual Care

Medicines: TCA = Tricyclic Antidepressants, SSRI = Selective Serotonin Reuptake Inhibitor

Therapies: ASSIST = Alcohol, Smoking, and Substance Involvement Screening Test, HAP = Healthy Activity Program, IPT = Interpersonal Therapy, CBT = Cognitive Behavioral Therapy, CPT = Cognitive Processing Therapy, PST = Problem-solving Therapy, CETA = Common Elements Treatment Approach, MI = Motivational Interviewing, MET = Motivational Enhancement Therapy, BI = Brief Intervention, SA= Simple Advice, CCM = Collaborative Care Model

Economic evaluation: QALY = Quality-Adjusted Life Year

**REFERENCES**

1. Al-Faris E, Al-Subaie A, Khoja T, Al-Ansary L, Abdul-Raheem F, Al-Hamdan N, et al. Training primary health care physicians in Saudi Arabia to recognize psychiatric illness. Acta Psychiatr Scand [Internet]. 1997 Dec [cited 2018 Oct 31];96(6):439–44. Available from: http://www.ncbi.nlm.nih.gov/pubmed/9421340

2. Ali BS, Rahbar MH, Naeem S, Gul A, Mubeen S, Iqbal A. The effectiveness of counseling on anxiety and depression by minimally trained counselors: a randomized controlled trial. Am J Psychother [Internet]. 2003 Jul [cited 2018 Oct 11];57(3):324–36. Available from: http://psychotherapy.psychiatryonline.org/doi/10.1176/appi.psychotherapy.2003.57.3.324

3. Ali NS, Ali BS, Azam IS, Khuwaja AK. Effectiveness of counseling for anxiety and depression in mothers of children ages 0-30 months by community workers in Karachi, Pakistan: a quasi experimental study. BMC Psychiatry [Internet]. 2010 [cited 2018 Oct 4];10:57. Available from: https://www.ncbi.nlm.nih.gov/pmc/articles/PMC2912800/

4. Alvarado R, Rojas G. El programa nacional para el diagnóstico y tratamiento de depresión en atención primaria: Una evaluación necesaria. Rev Med Chil. 2011;139(5):592–9.

5. Alvarado R, Vega J, Sanhueza G, María Y, Muñoz G, De Citar F. Evaluación del Programa para la Detección, Diagnóstico y Tratamiento Integral de la Depresión en atención primaria, en Chile [Internet]. Vol. 18, Public Health. 2005 [cited 2019 Jul 23]. Available from: https://rcweb.dartmouth.edu/~bartelss/pdf/team1/3211441679/alvarado 2005.pdf

6. Antini C, Depresión I De. Mejoría de la calidad de vida en personas atendidas en el “ Programa para la Detección , Diagnóstico y Tratamiento Integral de la Depresión en Atención Primaria ”, en Chile. 2008;72(2):142–9.

7. Araya R, Rojas G, Fritsch R, Gaete J, Rojas M, Simon G, et al. Treating depression in primary care in low-income women in Santiago, Chile: a randomised controlled trial. Lancet (London, England) [Internet]. 2003 Mar 22 [cited 2018 Nov 9];361(9362):995–1000. Available from: https://linkinghub.elsevier.com/retrieve/pii/S0140673603128255

8. Araya R, Flynn T, Rojas G, Fritsch R, Simon G. Cost-effectiveness of a primary care treatment program for depression in low-income women in Santiago, Chile. Am J Psychiatry [Internet]. 2006 Aug [cited 2018 Nov 9];163(8):1379–87. Available from: http://psychiatryonline.org/doi/abs/10.1176/ajp.2006.163.8.1379

9. Assanangkornchai S, Nima P, McNeil EB, Edwards JG. Comparative trial of the WHO ASSIST-linked brief intervention and simple advice for substance abuse in primary care. Asian J Psychiatr [Internet]. 2015;18(2015):75–80. Available from: http://dx.doi.org/10.1016/j.ajp.2015.09.003

10. Baker-Henningham H, Powell C, Walker S, Grantham S. The effect of early stimulation on maternal depression: a cluster randomised controlled trial. Arch Dis Child [Internet]. 2005 [cited 2019 Jul 23];90:1230–4. Available from: www.archdischild.com

11. Berbesi, Dedsy; Segura, Angela; Torres Y. Evaluación de un programa para el tratamiento de la depresión en Antioquia , Colombia , 2007 Evaluation of a program for the treatment of depression in Antioquia ,. Rev Fac Nac Salud Publica. 2010;28(1):48–55.

12. Buttorff C, Hock R, Weiss H, Naik S, Araya R, Kirkwood B, et al. Economic evaluation of a task-shifting intervention for common mental disorders in India. Bull World Health Organ [Internet]. 2012 Nov 1 [cited 2018 Oct 3];90(11):813–21. Available from: http://www.ncbi.nlm.nih.gov/pubmed/23226893

13. Chen S, Conwell Y, He J, Lu N, Wu J. Depression care management for adults older than 60 years in primary care clinics in urban China: a cluster-randomised trial. The lancet Psychiatry. 2015 Apr;2(4):332–9.

14. Chibanda D, Mesu P, Kajawu L, Cowan F, Araya R, Abas MA. Problem-solving therapy for depression and common mental disorders in Zimbabwe: piloting a task-shifting primary mental health care intervention in a population with a high prevalence of people living with HIV. BMC Public Health [Internet]. 2011 Oct 26 [cited 2018 Oct 15];11:828. Available from: http://www.ncbi.nlm.nih.gov/pubmed/22029430

15. Chibanda D, Shetty AK, Tshimanga M, Woelk G, Stranix-Chibanda L, Rusakaniko S. Group problem-solving therapy for postnatal depression among HIV-positive and HIV-negative mothers in Zimbabwe. J Int Assoc Provid AIDS Care [Internet]. 2014 [cited 2018 Oct 15];13(4):335–41. Available from: http://www.ncbi.nlm.nih.gov/pubmed/25513030

16. Chibanda D, Weiss H, Verhey R, Simms V, Rusakaniko S, Chingono A, et al. Effect of a Primary Care–Based Psychological Intervention on Symptoms of Common Mental Disorders in Zimbabwe A Randomized Clinical Trial. JAMA. 2016;316(24):2618–26.

17. Chisholm D, Sekar K, Kumar KK, Saeed K, James S, Mubbashar M, et al. Integration of mental health care into primary care. Demonstration cost-outcome study in India and Pakistan. Br J Psychiatry [Internet]. 2000 Jun [cited 2018 Oct 16];176:581–8. Available from: http://www.ncbi.nlm.nih.gov/pubmed/10974966

18. Chowdhary N, Anand A, Dimidjian S, Shinde S, Weobong B, Balaji M, et al. The Healthy Activity Program lay counsellor delivered treatment for severe depression in India: systematic development and randomised evaluation. Br J Psychiatry [Internet]. 2016 Apr [cited 2018 Oct 15];208(4):381–8. Available from: http://www.ncbi.nlm.nih.gov/pubmed/26494875

19. del Carmen Lara-Muñoz M, Robles-García R, Orozco R, Real T, Chisholm D, Medina-Mora ME. Estudio de costo-efectividad del tratamiento de la depresión en México. Salud Ment. 2010;33(4):301–8.

20. Fairall LR, Folb N, Timmerman V, Lombard C, Steyn K, Bachmann MO, et al. Educational Outreach with an Integrated Clinical Tool for Nurse-Led Non- communicable Chronic Disease Management in Primary Care in South Africa : A Pragmatic Cluster Randomised Controlled Trial. PLoS Med. 2016;

21. Fritsch R, Araya R, Solís J, Montt E, Pilowsky D, Rojas G. Un ensayo clínico aleatorizado de farmacoterapia con monitorización telefónica para mejorar el tratamiento de la depresión en la atención primaria en Santiago, Chile. Rev Med Chil [Internet]. 2007 May [cited 2018 Nov 2];135(5):587–95. Available from: http://www.scielo.cl/scielo.php?script=sci_arttext&pid=S0034-98872007000500006&lng=en&nrm=iso&tlng=en

22. García-Peña C, Vázquez-Estupiñan F, Avalos-Pérez F, Viridiana L, Jiménez R, Sánchez-Garcia S, et al. Clinical effectiveness of group cognitive-behavioural therapy for depressed older people in primary care: A randomised controlled trial. Salud Ment [Internet]. 2015 [cited 2018 Nov 15];38(1):33–9. Available from: https://pdfs.semanticscholar.org/c58f/b48dfe715784f07091db3b8663665e553062.pdf

23. Gómez-restrepo C, Peñaranda AB, Fabian J, Laverde G, Tamayo N, Sánchez DR. Exactitud en el diagnóstico de depresión por médicos de atención primaria después de una intervención educativa *. Rev Colomb Psiquiatr. 2007;XXXVI(3):439–50.

24. Gureje O, Chisholm D, Kola L, Lasebikan V, Saxena S. Cost-effectiveness of an essential mental health intervention package in Nigeria. World Psychiatry [Internet]. 2007 Feb [cited 2018 Oct 3];6(1):42–8. Available from: http://www.ncbi.nlm.nih.gov/pubmed/17342226

25. Husain N, Chaudhry N, Fatima B, Husain M, Amin R, Chaudhry IB, et al. Antidepressant and group psychosocial treatment for depression: A rater blind exploratory RCT from a low income country. Behav Cogn Psychother. 2014;42(6):693–705.

26. James S, Chisholm D, Murthy RS, Kumar KK, Sekar K, Saeed K, et al. Demand for, access to and use of community mental health care: lessons from a demonstration project in India and Pakistan. Int J Soc Psychiatry [Internet]. 2002 Sep 27 [cited 2018 Oct 15];48(3):163–76. Available from: http://journals.sagepub.com/doi/10.1177/002076402128783217

27. Jenkins R, Othieno C, Okeyo S, Kaseje D, Aruwa J, Oyugi H, et al. Short structured general mental health in service training programme in Kenya improves patient health and social outcomes but not detection of mental health problems - a pragmatic cluster randomised controlled trial. Int J Ment Health Syst [Internet]. 2013 Nov 5 [cited 2018 Oct 8];7(1):25. Available from: http://www.ncbi.nlm.nih.gov/pubmed/24188964

28. Kauye F, Jenkins R, Rahman A. Training primary health care workers in mental health and its impact on diagnoses of common mental disorders in primary care of a developing country, Malawi: a cluster-randomized controlled trial. Psychol Med [Internet]. 2014 Feb [cited 2018 Nov 9];44(03):657–66. Available from: http://www.journals.cambridge.org/abstract_S0033291713001141

29. L’engle KL, Mwarogo P, Kingola N, Sinkele W, Weiner DH. A Randomized Controlled Trial of a Brief Intervention to Reduce Alcohol Use Among Female Sex Workers in Mombasa, Kenya. J Acquir Immune Defic Syndr [Internet]. 2014 [cited 2019 Jul 23];67:446–53. Available from: www.jaids.com

30. Lima AFB da S, Fleck MP de A. Quality of life, diagnosis, and treatment of patients with major depression: a prospective cohort study in primary care. Rev Bras Psiquiatr [Internet]. 2011 Mar 11 [cited 2018 Oct 17];33(3):245–51. Available from: http://www.scielo.br/scielo.php?script=sci_arttext&pid=S1516-44462011000300007&lng=en&tlng=en

31. Malakouti SK, Nojomi M, Ahmadkhaniha HR, Hosseini M, Yekeh Fallah M, Mirzaei Khoshalani M. Integration of suicide prevention program into primary health care network: a field clinical trial in Iran. Med J Islam Repub Iran [Internet]. 2015 [cited 2018 Oct 17];29:208. Available from: http://www.ncbi.nlm.nih.gov/pubmed/26157726

32. Maulik PK, Kallakuri S, Devarapalli S, Vadlamani VK, Jha V, Patel A. Increasing use of mental health services in remote areas using mobile technology: a pre–post evaluation of the SMART Mental Health project in rural India. J Glob Health. 2017 Mar 21;7(1).

33. Mertens JR, Ward CL, Bresick GF, Broder T, Weisner CM. Effectiveness of nurse-practitioner-delivered brief motivational intervention for young adult alcohol and drug use in primary care in South Africa: a randomized clinical trial. Alcohol Alcohol [Internet]. 2014 [cited 2018 Oct 17];49(4):430–8. Available from: http://www.ncbi.nlm.nih.gov/pubmed/24899076

34. Nadkarni A, Weobong B, Weiss HA, McCambridge J, Bhat B, Katti B, et al. Counselling for Alcohol Problems (CAP), a lay counsellor-delivered brief psychological treatment for harmful drinking in men, in primary care in India: a randomised controlled trial. Lancet (London, England) [Internet]. 2017 [cited 2018 Oct 15];389(10065):186–95. Available from: http://www.ncbi.nlm.nih.gov/pubmed/27988144

35. Nakimuli-Mpungu E, Wamala K, Okello J, Alderman S, Odokonyero R, Mojtabai R, et al. Group support psychotherapy for depression treatment in people with HIV/AIDS in northern Uganda: a single-centre randomised controlled trial. Lancet HIV [Internet]. 2015 [cited 2019 Jul 23];2. Available from: www.thelancet.com/

36. Niemi M, Kiel S, Allebeck P, Hoan LT. Community-based intervention for depression management at the primary care level in Ha Nam Province, Vietnam: a cluster-randomised controlled trial. Trop Med Int Health [Internet]. 2016 May [cited 2018 Nov 9];21(5):654–61. Available from: http://doi.wiley.com/10.1111/tmi.12674

37. Noknoy S, Rangsin R, Saengcharnchai P, Tantibhaedhyangkul U, McCambridge J. RCT of effectiveness of motivational enhancement therapy delivered by nurses for hazardous drinkers in primary care units in Thailand. Alcohol Alcohol [Internet]. 2010 [cited 2018 Oct 8];45(3):263–70. Available from: http://www.ncbi.nlm.nih.gov/pubmed/20236990

38. Oladeji BD, Kola L, Abiona T, Montgomery AA, Araya R, Gureje O. A pilot randomized controlled trial of a stepped care intervention package for depression in primary care in Nigeria. BMC Psychiatry. 2015 May;15:96.

39. Papas RK, Sidle JE, Gakinya BN, Baliddawa JB, Martino S, Mwaniki MM, et al. Treatment outcomes of a stage 1 cognitive-behavioral trial to reduce alcohol use among human immunodeficiency virus-infected out-patients in western Kenya. Addiction [Internet]. 2011 Dec [cited 2018 Oct 15];106(12):2156–66. Available from: http://www.ncbi.nlm.nih.gov/pubmed/21631622

40. Patel V, Weiss HA, Chowdhary N, Naik S, Pednekar S, Chatterjee S, et al. Effectiveness of an intervention led by lay health counsellors for depressive and anxiety disorders in primary care in Goa, India (MANAS): a cluster randomised controlled trial. Lancet (London, England) [Internet]. 2010 Dec 18 [cited 2018 Oct 8];376(9758):2086–95. Available from: http://www.ncbi.nlm.nih.gov/pubmed/21159375

41. Patel V, Weiss HA, Chowdhary N, Naik S, Pednekar S, Chatterjee S, et al. Lay health worker led intervention for depressive and anxiety disorders in India: impact on clinical and disability outcomes over 12 months. Br J Psychiatry [Internet]. 2011 Dec [cited 2018 Nov 2];199(6):459–66. Available from: http://www.ncbi.nlm.nih.gov/pubmed/22130747

42. Patel V, Weobong B, Weiss HA, Anand A, Bhat B, Katti B, et al. The Healthy Activity Program (HAP), a lay counsellor-delivered brief psychological treatment for severe depression, in primary care in India: a randomised controlled trial. Lancet (London, England). 2017;389(10065):176–85.

43. Peltzer K, Naidoo P, Louw J, Matseke G, Zuma K, McHunu G, et al. Screening and brief interventions for hazardous and harmful alcohol use among patients with active tuberculosis attending primary public care clinics in South Africa: results from a cluster randomized controlled trial. BMC Public Health [Internet]. 2013 Jul 31 [cited 2018 Nov 22];13:699. Available from: http://www.ncbi.nlm.nih.gov/pubmed/23902931

44. Petersen I, Bhana A, Baillie K, MhaPP Research Programme Consortium. The feasibility of adapted group-based interpersonal therapy (IPT) for the treatment of depression by community health workers within the context of task shifting in South Africa. Community Ment Health J [Internet]. 2012 Jun 18 [cited 2018 Oct 22];48(3):336–41. Available from: http://link.springer.com/10.1007/s10597-011-9429-2

45. Petersen I, Fairall L, Egbe CO, Bhana A. Optimizing lay counsellor services for chronic care in South Africa: A qualitative systematic review. Patient Educ Couns [Internet]. 2014 [cited 2019 Jan 9];95:201–10. Available from: http://dx.doi.org/10.1016/j.pec.2014.02.001

46. Pradeep J, Isaacs A, Shanbag D, Selvan S, Srinivasan K. Enhanced care by community health workers in improving treatment adherence to antidepressant medication in rural women with major depression. Indian J Med Res [Internet]. 2014 Feb [cited 2018 Oct 16];139(2):236–45. Available from: http://www.ncbi.nlm.nih.gov/pubmed/24718398

47. Rahman A, Malik A, Sikander S, Roberts C, Creed F. Cognitive behaviour therapy-based intervention by community health workers for mothers with depression and their infants in rural Pakistan: a cluster-randomised controlled trial. Lancet [Internet]. 2008 [cited 2018 Oct 4];372(9642):902. Available from: https://www.ncbi.nlm.nih.gov/pmc/articles/PMC2603063/

48. Rahman A, Usman Hamdani S, Riaz Awan N, Bryant RA, Dawson KS, Firaz Khan M, et al. Effect of a Multicomponent Behavioral Intervention in Adults Impaired by Psychological Distress in a Conflict-Affected Area of Pakistan A Randomized Clinical Trial. JAMA [Internet]. 2016 [cited 2019 Jun 24];316(24):2609–17. Available from: https://rcweb.dartmouth.edu/~bartelss/pdf/team3/2053999932/Rahman-2016-Effect of a Multicomponent Behavio.pdf

49. Ramarumo M, Peltzer K, Khoza LB. Screening and brief intervention of alcohol problems in primary care in South Africa : A brief report. J Psychol Africa. 2016;26(1):78–80.

50. Rendall-Mkosi K, Morojele N, London L, Moodley S, Singh C, Girdler-brown B. A randomized controlled trial of motivational interviewing to prevent risk for an alcohol-exposed pregnancy in the Western Cape , South Africa. Addiction. 2012;108:725–32.

51. Rojas G, Fritsch R, Solis J, Jadresic E, Castillo C, González M, et al. Treatment of postnatal depression in low-income mothers in primary-care clinics in Santiago, Chile: a randomised controlled trial. Lancet [Internet]. 2007 Nov 10 [cited 2018 Nov 9];370(9599):1629–37. Available from: http://www.ncbi.nlm.nih.gov/pubmed/17993363

52. Rojas MG, Castro A, Guajardo V, Alvarado R, Isamit C, Fritsch R. Programa colaborativo a distancia para el tratamiento de la enfermedad depresiva. Rev Med Chil. 2014;142(9):1142–9.

53. Rotheram-Borus MJ, Tomlinson M, Roux I Le, Stein JA. Alcohol Use, Partner Violence, and Depression. Am J Prev Med [Internet]. 2015;49(5):715–25. Available from: http://dx.doi.org/10.1016/j.amepre.2015.05.004

54. Shidhaye R, Murhar V, Gangale S, Aldridge L, Shastri R, Parikh R, et al. The effect of VISHRAM, a grass-roots community-based mental health programme, on the treatment gap for depression in rural communities in India: a population-based study. The lancet Psychiatry [Internet]. 2017 Feb [cited 2018 Oct 17];4(2):128–35. Available from: https://linkinghub.elsevier.com/retrieve/pii/S2215036616304242

55. Siskind D, Araya R, Kim J. Cost-effectiveness of improved primary care treatment of depression in women in Chile. Br J Psychiatry. 2010;291–6.

56. Sorsdahl K, Petersen P. Feasibility and Preliminary Responses to a Screening and Brief Intervention Program for Maternal Mental Disorders Within the Context of Primary Care. Community Ment Health J [Internet]. 2015;962–9. Available from: http://dx.doi.org/10.1007/s10597-015-9853-9

57. Tomlinson M, Rotheram-Borus MJ, Le Roux IM, Youssef M, Nelson SH, Scheffler A, et al. Thirty-Six-Month Outcomes of a Generalist Paraprofessional Perinatal Home Visiting Intervention in South Africa on Maternal Health and Child Health and Development. [cited 2019 Jul 25]; Available from: https://clinicaltrials.gov/ct2/show/NCT00972699

58. Weiss WM, Murray LK, Abdulla G, Zangana S, Mahmooth Z, Kaysen D, et al. Community-based mental health treatments for survivors of torture and militant attacks in Southern Iraq: a randomized control trial. BMC Psychiatry [Internet]. 2015 [cited 2018 Nov 15]; Available from: http://tinyurl.com/CETA-Iraq-Protocol.
