# Supplementary material for: The effectiveness and cost-effectiveness of integrating mental health services in primary care in low- and middle-income countries: systematic review
Source: BJPsych Bull. 2021 Feb;45(1):40–52. doi: 10.1192/bjb.2020.35 (PMC8058938; doi:10.1192/bjb.2020.35)
Supplement: Supplementary file 1 [file S2056469420000352sup.zip › S2056469420000352sup001.docx]

**Supplementary Data** Search Strategy.

1. **Databases**

| Database | Platform | Years covered | Date conducted | # results |
| --- | --- | --- | --- | --- |
| Medline | PubMed | 1990-current | April 28, 2017 | 2520 |
| Embase | Elsevier | 1990-current | May 4, 2017 | 2927 |
| Web of Science | Thomson Reuters | 1990-current | April 28, 2017 | 5181 |
| Cochrane | Wiley | 1990-current  DSR: issue 4, Apr2017  Trials: issue 3, Mar2017  Methods: issue 3, July2012  EconEval: issue 2, 2017 | April 28, 2017 | 376  [DSR-31  Trials-339  Methods-2  EconEval-4] |
| *WHO Global Index Medicus | globalhealthlibrary.net | 1990-current | April 28, 2017 | 1254  [LILACS-775  WPRIM-356  IMEMR-61  IMSEAR-53  AIM-9] |
| PsycINFO | Proquest | 1990-current | April 28, 2017 | 1241 |
| Total |  |  |  | 13499 |
| Total with duplicates removed |  |  |  | 8786 |

*WHO Global Index Medicus search did not include LMIC concept

1. **PubMed (Medline)**

Run on April 28, 2017

| Search | Query | Items found |
| --- | --- | --- |
| #5 | (#1 AND #2 AND #3) Filters: Publication date from 1990/01/01 | 2520 |
| #4 | (#1 AND #2 AND #3) | 2638 |
| #3 | Depression[mesh] OR Depressive Disorder[Mesh] OR Alcohol-Related Disorders[mesh] OR Alcohol Drinking[Mesh] OR Depression[tiab] OR Depressed[tiab] OR Depressive[tiab] OR Mood[tiab] OR Psychological indicator[tiab] OR Emotional functioning[tiab] OR Mental functioning[tiab] OR Psychological status[tiab] OR Psychological coping[tiab] OR Binge drink*[tiab] OR Alcoholic*[tiab] OR Alcoholism[tiab] OR Alcohol use disorder[tiab] OR Alcohol misuse[tiab] OR Alcohol abuse[tiab] OR Alcohol dependence[tiab] OR Alcohol addiction[tiab] OR Ethanol use disorder[tiab] OR Ethanol misuse[tiab] OR Ethanol abuse[tiab] OR Ethanol dependence[tiab] OR Ethanol addiction[tiab] OR AOD[tiab] OR Underage drink*[tiab] OR Under-age drink* [tiab] | 604087 |
| #2 | “Delivery of Health Care, Integrated"[Mesh:NoExp] OR "Continuity of Patient Care"[Mesh:NoExp] OR "Transitional Care"[Mesh] OR Case Management[mesh] OR "Patient-Centered Care"[Mesh] OR Patient Care Management[mesh:noexp] OR "Comprehensive Health Care"[Mesh:NoExp] OR "Primary health care"[mesh:noexp] OR "Community health services"[mesh:noexp] OR "Community health nursing"[mesh] OR "Community mental health services"[mesh] OR "Community health workers"[mesh] OR "Home Care Services"[Mesh] OR Integrat*[tiab] OR collaborat*[tiab] OR Managed care[tiab] OR Care management[tiab] OR Shared care[tiab] OR Transmural care[tiab] OR Continuity of Patient Care[tiab] OR Continuity of Care[tiab] OR Case Management[tiab] OR Patient centered[tiab] OR Patient centred[tiab] OR People centered[tiab] OR People centred[tiab] OR Transitional care[tiab] OR Care transition*[tiab] OR Behavioral health home*[tiab] OR Behavioural health home*[tiab] OR mental health home*[tiab] OR Primary care[tiab] OR Community health [tiab] OR Community based care[tiab] OR Community based health*[tiab] OR Community based mental health[tiab] OR Community based management[tiab] OR Primary health care[tiab] OR Barefoot doctor*[tiab] OR Village health worker*[tiab] OR Home care[tiab] OR Lay health[tiab] OR (colocat*[tiab] AND (care[tiab] OR service*[tiab])) | 779534 |
| [#1](https://www-ncbi-nlm-nih-gov.dartmouth.idm.oclc.org/pubmed/advanced) | "Developing Countries"[Mesh] OR "Afghanistan"[Mesh] OR "Bahrain"[Mesh] OR "Iran"[Mesh] OR "Iraq"[Mesh] OR "Jordan"[Mesh] OR "Lebanon"[Mesh] OR "Oman"[Mesh] OR "Saudi Arabia"[Mesh] OR "Syria"[Mesh] OR "Turkey"[Mesh] OR "Yemen"[Mesh] OR "Europe, Eastern"[Mesh] OR "Africa"[Mesh] OR "Samoa"[Mesh] OR "Tonga"[Mesh] OR "South America"[Mesh] OR “Antigua and Barbuda"[Mesh] OR “Barbados"[Mesh] OR “Cuba"[Mesh] OR “Dominica"[Mesh] OR “Dominican Republic"[Mesh] OR “Grenada"[Mesh] OR “Haiti"[Mesh] OR “Jamaica"[Mesh] OR “Netherlands Antilles"[Mesh] OR “Puerto Rico"[Mesh] OR “Saint Kitts and Nevis"[Mesh] OR “Saint Lucia"[Mesh] OR “Saint Vincent and the Grenadines"[Mesh] OR “Trinidad and Tobago"[Mesh] OR "Transcaucasia"[Mesh] OR “Bangladesh"[Mesh] OR “Bhutan"[Mesh] OR “India"[Mesh] OR “Nepal"[Mesh] OR “Pakistan"[Mesh] OR “Sri Lanka” OR "Central America"[Mesh] OR "Melanesia"[Mesh] OR “Cambodia"[Mesh] OR “Indonesia"[Mesh] OR “Laos"[Mesh] OR “Malaysia"[Mesh] OR “Myanmar"[Mesh] OR “Philippines"[Mesh] OR “Thailand"[Mesh] OR “Timor-Leste"[Mesh] OR “Vietnam” OR "China"[Mesh:NoExp] OR "Macau"[Mesh] OR "Indian Ocean Islands"[Mesh] OR "Greece"[Mesh] OR "Portugal"[Mesh] OR "Asia, Central"[Mesh] OR "Micronesia"[Mesh] OR "Malta"[Mesh] OR "Korea"[Mesh] OR "Mongolia"[Mesh] OR "Atlantic Islands"[Mesh:NoExp] OR “USSR”[mesh] OR "Yugoslavia"[Mesh] OR ((developing[tiab] OR less developed[tiab] OR under developed[tiab] OR underdeveloped[tiab] OR transitional[tiab] OR middle income[tiab] OR low income[tiab] OR lower income[tiab]) AND (countr*[tiab] OR nation[tiab] OR nations[tiab] OR world[tiab] OR economy[tiab] OR economies[tiab])) OR ((low[tiab] OR lower[tiab]) AND (gdp[tiab] OR gnp[tiab] OR gross domestic[tiab] OR gross national[tiab])) OR lmic[tiab] OR lmics[tiab] OR Lamic[tiab] OR lamics[tiab] OR third world[tiab] OR lami countries[tiab] OR lami country[tiab] OR Afghanistan[tiab] OR Albania[tiab] OR Algeria[tiab] OR American Samoa[tiab] OR Angola[tiab] OR Antigua and Barbuda[tiab] OR Argentina[tiab] OR Armenia[tiab] OR Aruba[tiab] OR Azerbaijan[tiab] OR Bahrain[tiab] OR Bangladesh[tiab] OR Barbados[tiab] OR Belarus[tiab] OR Belize[tiab] OR Benin[tiab] OR Bhutan[tiab] OR Bolivia[tiab] OR Bosnia and Herzegovina[tiab] OR Botswana[tiab] OR Brazil[tiab] OR Bulgaria[tiab] OR Burkina Faso[tiab] OR Burundi[tiab] OR Cabo Verde[tiab] OR cape verde[tiab] OR Cambodia[tiab] OR Cameroon[tiab] OR Central African Republic[tiab] OR Chad[tiab] OR Chile[tiab] OR China[tiab] OR Colombia[tiab] OR Comoros[tiab] OR Congo[tiab] OR Costa Rica[tiab] OR Croatia[tiab] OR Cuba[tiab] OR Cyprus[tiab] OR Czech Republic[tiab] OR Czechoslovakia[tiab] OR Côte d'Ivoire[tiab] OR ivory coast[tiab] OR Côte dIvoire[tiab] OR Djibouti[tiab] OR Dominica[tiab] OR Dominican Republic[tiab] OR Ecuador[tiab] OR Egypt[tiab] OR El Salvador[tiab] OR Equatorial Guinea[tiab] OR Eritrea[tiab] OR Estonia[tiab] OR Ethiopia[tiab] OR Fiji[tiab] OR Gabon[tiab] OR Gambia[tiab] OR Georgia[tiab] OR Ghana[tiab] OR Gibraltar[tiab] OR Greece[tiab] OR Grenada[tiab] OR Guam[tiab] OR Guatemala[tiab] OR Guinea[tiab] OR Guinea-Bissau[tiab] OR Guyana[tiab] OR Haiti[tiab] OR Honduras[tiab] OR Hungary[tiab] OR India[tiab] OR Indonesia[tiab] OR Iran[tiab] OR Iraq[tiab] OR Isle of Man[tiab] OR Jamaica[tiab] OR Jordan[tiab] OR Kazakhstan[tiab] OR Kenya[tiab] OR Kiribati[tiab] OR Korea[tiab] OR Kosovo[tiab] OR Kyrgyz Republic[tiab] OR Kyrgyzstan[tiab] OR Kirgizstan[tiab] OR Lao PDR[tiab] OR laos[tiab] OR Latvia[tiab] OR Lebanon[tiab] OR Lesotho[tiab] OR Liberia[tiab] OR Libya[tiab] OR Lithuania[tiab] OR Macao[tiab] OR Macedonia[tiab] OR Madagascar[tiab] OR Malawi[tiab] OR Malaysia[tiab] OR Maldives[tiab] OR Mali[tiab] OR Malta[tiab] OR Marshall Islands[tiab] OR Mauritania[tiab] OR Mauritius[tiab] OR Mayotte[tiab] OR Mexico[tiab] OR Micronesia[tiab] OR Moldova[tiab] OR Mongolia[tiab] OR Montenegro[tiab] OR Morocco[tiab] OR Mozambique[tiab] OR Myanmar[tiab] OR Namibia[tiab] OR Nepal[tiab] OR Netherlands Antilles[tiab] OR New Caledonia[tiab] OR Nicaragua[tiab] OR Niger[tiab] OR Nigeria[tiab] OR Mariana Islands[tiab] OR Oman[tiab] OR Pakistan[tiab] OR Palau[tiab] OR Panama[tiab] OR Papua New Guinea[tiab] OR Paraguay[tiab] OR Peru[tiab] OR Philippines[tiab] OR Poland[tiab] OR Portugal[tiab] OR Puerto Rico[tiab] OR Romania[tiab] OR Russian Federation[tiab] OR russia[tiab] OR Rwanda[tiab] OR Samoa[tiab] OR Saudi Arabia[tiab] OR Senegal[tiab] OR Serbia[tiab] OR Seychelles[tiab] OR Sierra Leone[tiab] OR Slovak Republic[tiab] OR slovakia[tiab] OR Slovenia[tiab] OR Solomon Islands[tiab] OR Somalia[tiab] OR South Africa[tiab] OR South Sudan[tiab] OR Sri Lanka[tiab] OR Kitts and Nevis[tiab] OR st Kitts[tiab] OR saint kitts[tiab] OR St Lucia[tiab] OR saint lucia[tiab] OR Grenadines[tiab] OR Sudan[tiab] OR Suriname[tiab] OR Swaziland[tiab] OR Syrian Arab Republic[tiab] OR syria[tiab] OR São Tomé[tiab] OR Tajikistan[tiab] OR Tanzania[tiab] OR Thailand[tiab] OR Timor-Leste[tiab] OR Togo[tiab] OR Tonga[tiab] OR Trinidad[tiab] OR Tobago[tiab] OR Tunisia[tiab] OR Turkey[tiab] OR Turkmenistan[tiab] OR Tuvalu[tiab] OR USSR[tiab] OR Uganda[tiab] OR Ukraine[tiab] OR Uruguay[tiab] OR Uzbekistan[tiab] OR Vanuatu[tiab] OR Venezuela[tiab] OR Vietnam[tiab] OR West Bank[tiab] OR Gaza[tiab] OR Yemen[tiab] OR Yugoslavia[tiab] OR Zambia[tiab] OR Zimbabwe[tiab] | 1518101 |

1. **EMBASE** (Elsevier)

Run on May 4, 2017

‘depression'/exp OR 'major depression'/exp OR 'alcoholism'/exp OR 'drinking behavior'/exp OR depression:ab,ti OR depressed:ab,ti OR depressive:ab,ti OR mood:ab,ti OR 'psychological indicator':ab,ti OR 'emotional functioning':ab,ti OR 'mental functioning':ab,ti OR 'psychological status':ab,ti OR 'psychological coping':ab,ti OR 'binge drink*':ab,ti OR alcoholic*:ab,ti OR alcoholism:ab,ti OR 'alcohol use disorder':ab,ti OR 'alcohol misuse':ab,ti OR 'alcohol abuse':ab,ti OR 'alcohol dependence':ab,ti OR 'alcohol addiction':ab,ti OR 'ethanol use disorder':ab,ti OR 'ethanol misuse':ab,ti OR 'ethanol abuse':ab,ti OR 'ethanol dependence':ab,ti OR 'ethanol addiction':ab,ti OR aod:ab,ti OR 'underage drink*':ab,ti OR 'under-age drink*':ab,ti AND [1990-2017]/py

**AND**

'integrated health care system'/de OR 'transitional care'/exp OR 'case management'/exp OR 'primary health care'/exp OR 'community care'/exp OR 'community health nursing'/exp OR 'home care'/exp OR 'health auxiliary'/exp OR integrat*:ab,ti OR collaborat*:ab,ti OR 'managed care':ab,ti OR 'care management':ab,ti OR 'shared care':ab,ti OR 'transmural care':ab,ti OR 'continuity of patient care':ab,ti OR 'continuity of care':ab,ti OR 'case management':ab,ti OR 'patient centered':ab,ti OR 'patient centred':ab,ti OR 'people centered':ab,ti OR 'people centred':ab,ti OR 'transitional care':ab,ti OR 'care transition':ab,ti OR 'behavioral health home':ab,ti OR 'behavioural health home':ab,ti OR 'mental health home':ab,ti OR 'primary care':ab,ti OR 'community health':ab,ti OR 'community based care':ab,ti OR 'community based health':ab,ti OR 'community based mental health':ab,ti OR 'community based management':ab,ti OR 'primary health care':ab,ti OR 'barefoot doctor*':ab,ti OR 'village health worker':ab,ti OR 'home care':ab,ti OR 'lay health':ab,ti OR (colocat* AND (care:ab,ti OR service*)) AND [1990-2017]/py

**AND**

'developing country'/exp AND [1990-2017]/py OR developing:ab,ti OR 'less developed':ab,ti OR 'under developed':ab,ti OR underdeveloped:ab,ti OR transitional:ab,ti OR 'middle income':ab,ti OR 'low income':ab,ti OR 'lower income':ab,ti AND (countr*:ab,ti OR nation:ab,ti OR nations:ab,ti OR world:ab,ti OR economy:ab,ti OR economies:ab,ti) AND [1990-2017]/py OR low:ab,ti OR lower:ab,ti AND (gdp:ab,ti OR gnp:ab,ti OR 'gross domestic':ab,ti OR 'gross national':ab,ti) OR lmic:ab,ti OR lmics:ab,ti OR lamic:ab,ti OR lamics:ab,ti OR 'third world':ab,ti OR 'lami countries':ab,ti OR 'lami country':ab,ti AND [1990-2017]/py OR 'afghanistan'/de OR afghanistan:ab,ti OR 'bahrain'/de OR bahrain:ab,ti OR 'iran'/de OR iran:ab,ti OR 'iraq'/de OR iraq:ab,ti OR 'jordan'/de OR jordan:ab,ti OR 'lebanon'/de OR lebanon:ab,ti OR 'oman'/de OR oman:ab,ti OR 'saudi arabia'/de OR 'saudi arabia':ab,ti OR 'syrian arab republic'/de OR syria:ab,ti OR 'turkey (republic)'/de OR turkey:ab,ti OR 'yemen'/de OR yemen:ab,ti OR 'eastern europe'/de OR 'eastern europe':ab,ti OR 'africa'/de OR africa:ab,ti OR 'samoa'/de AND samoa:ab,ti OR 'tonga'/de OR tonga:ab,ti OR 'south america'/de OR 'south america':ab,ti OR 'antigua and barbuda'/de OR 'antigua and barbuda':ab,ti OR 'barbados'/de OR barbados:ab,ti OR 'cuba'/de OR cuba:ab,ti OR 'dominica'/de OR dominica:ab,ti OR 'dominican republic'/de OR 'dominican republic':ab,ti OR 'grenada'/de OR grenada:ab,ti OR 'haiti'/de OR haiti:ab,ti OR 'jamaica'/de OR jamaica:ab,ti OR 'netherlands antilles'/de OR 'netherlands antilles':ab,ti OR 'puerto rico'/de OR 'puerto rico':ab,ti OR 'saint kitts and nevis'/de OR 'saint kitts and nevis':ab,ti OR 'saint lucia'/de OR 'saint lucia':ab,ti AND [1990-2017]/py OR 'saint vincent and the grenadines'/de OR 'saint vincent and the grenadines':ab,ti OR 'trinidad and tobago'/de OR 'trinidad and tobago':ab,ti OR transcaucasia:ab,ti OR 'bangladesh'/de OR 'bangladesh':ab,ti OR 'bhutan'/de OR bhutan:ab,ti OR 'india'/de OR india:ab,ti OR 'nepal'/de OR nepal:ab,ti OR 'pakistan'/de OR pakistan:ab,ti OR 'sri lanka'/de OR 'sri lanka':ab,ti OR 'central america'/de OR 'central america':ab,ti OR 'melanesia'/de OR melanesia:ab,ti OR 'cambodia'/de OR 'cambodia':ab,ti OR 'indonesia'/de OR 'indonesia':ab,ti OR 'laos':de OR 'laos':ab,ti OR 'malaysia'/de OR 'malaysia':ab,ti OR 'myammar':de OR myanmar:ab,ti OR 'phillipines' OR 'phillipines':ab,ti OR 'thailand'/de OR thailand:ab,ti OR 'timor-leste'/de OR 'timor-leste':ab,ti OR 'viet nam'/de OR 'viet nam':ab,ti OR 'china'/de OR china:ab,ti OR 'macao'/de OR macao:ab,ti OR 'indian ocean islands':ab,ti OR 'greece'/de OR 'greece':ab,ti OR 'portugal'/de OR portugal:ab,ti OR 'central asia':ab,ti OR 'federated states of micronesia'/de OR micronesia:ab,ti OR 'malta'/de OR malta:ab,ti OR 'korea'/de OR korea:ab,ti OR 'mongolia'/de OR mongolia:ab,ti OR 'atlantic islands'/de OR 'atlantic islands':ab,ti OR 'ussr'/exp OR 'ussr':ab,ti OR 'yugoslavia'/de OR 'yugoslavia':ab,ti AND [1990-2017]/py OR 'albania'/de OR albania:ab,ti OR 'algeria'/de OR algeria:ab,ti OR 'american samoa':de OR 'american samoa':ab,ti OR 'angola'/de OR angola:ab,ti OR 'argentina'/de OR argentina:ab,ti OR 'armenia'/de OR armenia:ab,ti OR 'aruba'/de OR aruba:ab,ti OR 'azerbaijan'/de OR azerbaijan:ab,ti OR 'bahrain'/de OR bahrain:ab,ti OR 'belarus'/de OR belarus:ab,ti OR 'belize'/de OR belize:ab,ti OR 'benin'/de OR benin:ab,ti OR 'bhutan'/de OR bhutan:ab,ti OR 'bolivia'/de OR bolivia:ab,ti OR 'bosnia and herzegovina'/de OR 'bosnia and herzegovina':ab,ti OR 'botswana'/de OR botswana:ab,ti OR 'brazil'/de OR brazil:ab,ti OR 'bulgaria'/de OR bulgaria:ab,ti OR 'burkina faso'/de OR 'burkina faso':ab,ti OR 'burundi'/de OR burundi;ab,ti OR 'cabo verde' OR 'cabo verde':ab,ti OR 'cape verde'/de OR 'cape verde':ab,ti OR 'cambodia'/de OR cambodia:ab,ti AND [1990-2017]/py OR 'cameroon'/de OR cameroon:ab,ti OR 'central african republic'/de OR 'central african republic':ab,ti OR 'chad'/de OR chad:ab,ti OR 'chile'/de OR 'chile':ab,ti OR 'colombia'/de OR colombia:ab,ti OR 'comoros'/de OR comoros:ab,ti OR 'congo'/de OR congo:ab,ti OR 'costa rica'/de OR 'costa rica':ab,ti OR 'croatia'/de OR croatia:ab,ti OR 'cyprus'/de OR cyprus:ab,ti OR 'czech republic'/de OR 'czech republic':ab,ti OR 'czechoslovakia'/de OR 'czechoslovakia':ab,ti OR 'cote' NEAR/2 'ivoire' OR ('cote' NEAR/2 'ivoire'):ab,ti OR 'ivory coast':ab,ti OR 'djibouti'/de OR djibouti:ab,ti OR 'ecuador'/de OR ecuador:ab,ti OR 'egypt'/de OR egypt:ab,ti OR 'el salvador':de OR 'el salvador':ab,ti OR 'equatorial guinea'/de OR 'equatorial guinea':ab,ti OR 'eritrea'/de OR eritrea:ab,ti OR 'estonia'/de OR estonia:ab,ti OR 'ethiopia'/de OR ethiopia:ab,ti OR 'fiji'/de OR fiji:ab,ti OR 'gabon'/de OR gabon:ab,ti OR 'gambia'/de OR gambia:ab,ti OR 'georgia(republic)'/de OR georgia:ab,ti OR 'ghana'/de OR ghana:ab,ti OR 'gibraltar'/de OR gibraltar:ab,ti OR 'grenada'/de OR grenada:ab,ti AND [1990-2017]/py OR 'ivory coast':ab,ti OR 'djibouti'/de OR djibouti:ab,ti OR 'ecuador'/de OR ecuador:ab,ti OR 'egypt'/de OR egypt:ab,ti OR 'el salvador':de OR 'el salvador':ab,ti OR 'equatorial guinea'/de OR 'equatorial guinea':ab,ti OR 'eritrea'/de OR eritrea:ab,ti OR 'estonia'/de OR estonia:ab,ti OR 'ethiopia'/de OR ethiopia:ab,ti OR 'fiji'/de OR fiji:ab,ti OR 'gabon'/de OR gabon:ab,ti OR 'gambia'/de OR gambia:ab,ti OR 'georgia(republic)'/de OR georgia:ab,ti OR 'ghana'/de OR ghana:ab,ti OR 'gibraltar'/de OR gibraltar:ab,ti OR 'grenada'/de OR grenada:ab,ti OR 'guam'/de OR guam:ab,ti OR 'guatemala'/de OR guatemala:ab,ti OR 'guinea'/de OR guinea:ab,ti OR 'guinea-bissau'/de OR 'guinea bissau':ab,ti OR 'guyana'/de OR guyana:ab,ti OR 'honduras'/de OR honduras:ab,ti OR 'hungary'/de OR hungary:ab,ti OR 'indonesia'/de OR indonesia:ab,ti OR 'isle of man'/de OR 'isle of man':ab,ti AND [1990-2017]/py OR 'kazakhstan'/de OR kazakhstan:ab,ti OR 'kenya'/de OR kenya:ab,ti OR 'kiribati'/de OR kiribati:ab,ti OR 'korea'/de OR korea:ab,ti OR 'kosovo'/de OR kosovo:ab,ti OR 'kyrgyzstan'/de OR kyrgyzstan:ab,ti OR kirgizstan:ab,ti OR 'laos'/de OR laos:ab,ti OR 'latvia'/de OR latvia:ab,ti OR 'lebanon'/de OR lebanon:ab,ti OR 'lesotho'/de OR lesotho:ab,ti OR 'liberia'/de OR liberia:de OR liberia:ab,ti OR 'libyan arab jamahiriya'/de OR libya:ab,ti OR 'lithuania'/de OR lithuania:ab,ti OR 'macedonia(republic)'/de OR macedonia:ab,ti OR 'madagascar'/de OR 'madagascar':ab,ti OR 'malawi'/de OR malawi:ab,ti OR 'malaysia'/de OR malaysia:ab,ti OR 'maldives'/de OR maldives:ab,ti OR 'mali'/de OR mali:ab,ti OR 'malta'/de OR malta:ab,ti AND [1990-2017]/py OR 'marshall islands'/de OR 'marshall islands':ab,ti OR 'mauritania'/de OR mauritania:ab,ti OR 'mayotte'/de OR mayotte:ab,ti OR 'mexico'/de OR mexico:ab,ti OR 'federated states of micronesia'/de OR micronesia:ab,ti OR 'moldova'/de OR moldova:ab,ti OR 'mongolia'/de OR mongolia:ab,ti OR 'montenegro (republic)'/de OR montenegro:ab,ti OR 'morocco'/de OR morocco:ab,ti OR 'mozambique'/de OR mozambique:ab,ti OR 'myanmar'/de OR myanmar:ab,ti OR 'namibia'/de OR namibia:ab,ti OR 'nepal'/de OR nepal:ab,ti OR 'new caledonia'/de OR 'new caledonia':ab,ti OR 'nicaragua':de OR nicaragua:ab,ti OR 'niger'/de OR niger:ab,ti OR 'nigeria'/de OR nigeria:ab,ti OR 'mariana islands':ab,ti OR 'oman'/de OR oman:ab,ti OR 'pakistan'/de OR pakistan:ab,ti OR 'palau'/de OR palau:ab,ti OR 'panama'/de OR panama:ab,ti OR 'papua new guinea'/de OR 'papua new guinea':ab,ti OR 'paraguay'/de OR paraguay:ab,ti OR 'peru'/de OR peru:ab,ti OR 'poland'/de OR poland:ab,ti OR 'portugal'/de OR portugal:ab,ti OR 'puerto rico'/de OR 'puerto rico':ab,ti OR 'romania'/de OR romania:ab,ti OR 'russian federation'/de OR 'russian federation':ab,ti OR russia:ab,ti OR 'rwanda'/de OR rwanda:ab,ti OR 'samoa'/de OR samoa:ab,ti OR saudi AND 'arabia'/de OR 'saudi arabia':ab,ti OR 'senegal'/de OR senegal:ab,ti AND [1990-2017]/py OR 'serbia'/de OR serbia:ab,ti OR 'seychelles'/de OR seychelles:ab,ti OR 'sierra leone'/de OR 'sierra leone':ab,ti OR 'slovakia'/de OR slovakia:ab,ti OR 'slovenia'/de OR slovenia:ab,ti OR 'solomon islands'/de OR 'solomon islands':ab,ti OR 'somalia'/de OR somalia:ab,ti OR 'south africa'/de OR 'south africa':ab,ti OR 'south sudan'/de OR 'south sudan':ab,ti OR sri AND lanka OR 'sri lanka':ab,ti OR 'st kitts':ab,ti OR 'saint kitts':ab,ti OR 'st lucia':ab,ti OR 'saint lucia':ab,ti OR grenadines:ab,ti OR 'sudan':ab,ti OR 'suriname'/de OR suriname:ab,ti OR 'swaziland'/de OR swaziland:ab,ti OR syria:ab,ti OR 'sao tome and principe'/de OR 'sao tome':ab,ti OR 'tajikistan'/de OR tajikistan:ab,ti OR 'tanzania'/de OR tanzania:ab,ti OR 'togo'/de OR togo:ab,ti AND [1990-2017]/py OR 'tonga'/de OR tonga:ab,ti OR 'tunisia'/de OR tunisia:ab,ti OR 'turkmenistan'/de OR turkmenistan:ab,ti OR 'tuvalu'/de OR tuvalu:ab,ti OR 'uganda'/de OR 'uganda':ab,ti OR 'ukraine'/de OR 'ukraine':ab,ti OR 'uruguay'/de OR 'uruguay':ab,ti OR 'uzbekistan'/de OR 'uzbekistan':ab,ti OR 'vanuatu'/de OR vanuatu:ab,ti OR 'venezuela'/de OR venezuela:ab,ti AND [1990-2017]/py OR 'west bank':ab,ti AND [1990-2017]/py OR gaza:ab,ti OR 'yemen'/de OR yemen:ab,ti OR 'zambia'/de OR zambia:ab,ti OR 'zimbabwe'/de OR zimbabwe:ab,ti AND [1990-2017]/py

Results = 2927

1. **Web of Science** (Thomson Reuters)

Databases searched: Web of Science Core Collection, Korean Journal Database, Russian Citation Index, SciELO Citation Index

Run on April 28, 2017

| **Set** | **Results** |  |
| --- | --- | --- |
| # 6 | [**5,181**](http://apps.webofknowledge.com.dartmouth.idm.oclc.org/summary.do?product=UA&doc=1&qid=11&SID=4AfsjzUEVd6uSBMYFVU&search_mode=AdvancedSearch&update_back2search_link_param=yes) | #5  *Timespan=1990-2017*  *Search language=Auto* |
| # 5 | [**5,259**](http://apps.webofknowledge.com.dartmouth.idm.oclc.org/summary.do?product=UA&doc=1&qid=7&SID=4AfsjzUEVd6uSBMYFVU&search_mode=CombineSearches&update_back2search_link_param=yes) | #3 AND #2 AND #1  **Refined by:** **Databases:** ( WOS OR SCIELO OR RSCI OR KJD )  *Timespan=All years*  *Search language=Auto* |
| # 4 | [**5,912**](http://apps.webofknowledge.com.dartmouth.idm.oclc.org/summary.do?product=UA&doc=1&qid=6&SID=4AfsjzUEVd6uSBMYFVU&search_mode=CombineSearches&update_back2search_link_param=yes) | #3 AND #2 AND #1  *Timespan=All years*  *Search language=Auto* |
| # 3 | [**1,004,291**](http://apps.webofknowledge.com.dartmouth.idm.oclc.org/summary.do?product=UA&doc=1&qid=5&SID=4AfsjzUEVd6uSBMYFVU&search_mode=GeneralSearch&update_back2search_link_param=yes) | **TOPIC:** (“Depression” OR “Depressed” OR “Depressive” OR “Mood” OR “Psychological indicator” OR “Emotional functioning” OR “Mental functioning” OR “Psychological status” OR “Psychological coping” OR “Binge drink*” OR “Alcoholic*” OR “Alcoholism” OR “Alcohol use disorder” OR “Alcohol misuse” OR “Alcohol abuse” OR “Alcohol dependence” OR “Alcohol addiction” OR “Ethanol use disorder” OR “Ethanol misuse” OR “Ethanol abuse” OR “Ethanol dependence” OR “Ethanol addiction” OR “AOD” OR “Underage drink*” OR “Under-age drink*”)  *Timespan=All years*  *Search language=Auto* |
| # 2 | [**2,433,143**](http://apps.webofknowledge.com.dartmouth.idm.oclc.org/summary.do?product=UA&doc=1&qid=4&SID=4AfsjzUEVd6uSBMYFVU&search_mode=GeneralSearch&update_back2search_link_param=yes) | **TOPIC:** (“Integrat*” OR “Collaborat*” OR “Managed care” OR “Care management” OR “Shared care” OR “Transmural care” OR “Continuity of Patient Care” OR “Continuity of Care” OR “Case Management” OR “Patient centered” OR “Patient centred” OR “People centered” OR “People centred” OR “Transitional care” OR “Care transition*” OR “Behavioral health home*” OR “Behavioural health home*” OR “Mental health home*” OR “Primary care” OR “Community health” OR “Community based care” OR “Community based health*” OR “Community based mental health” OR “Community based management” OR “Primary health care” OR “Barefoot doctor*” OR “Village health worker*” OR “Home care” OR “Lay health”) *OR* **TOPIC:** ((“colocat*” NEAR/4 (“care” OR “service*”)))  *Timespan=All years*  *Search language=Auto* |
| # 1 | [**4,534,491**](http://apps.webofknowledge.com.dartmouth.idm.oclc.org/summary.do?product=UA&doc=1&qid=1&SID=4AfsjzUEVd6uSBMYFVU&search_mode=GeneralSearch&update_back2search_link_param=yes) | **TOPIC:** (“Afghanistan” OR “Albania” OR “Algeria” OR “American Samoa” OR “Angola” OR “Antigua” and “Barbuda” OR “Argentina” OR “Armenia” OR “Aruba” OR “Azerbaijan” OR “Bahrain” OR “Bangladesh” OR “Barbados” OR “Belarus” OR “Belize” OR “Benin” OR “Bhutan” OR “Bolivia” OR “Bosnia” and “Herzegovina” OR “Botswana” OR “Brazil” OR “Bulgaria” OR “Burkina Faso” OR “Burundi” OR “Cabo Verde” OR “Cape Verde” OR Cambodia OR Cameroon OR “Central African Republic” OR “Chad” OR “Chile” OR “China” OR “Colombia” OR “Comoros” OR “Congo” OR “Costa Rica” OR “Croatia” OR “Cuba” OR “Cyprus” OR “Czech Republic” OR “Czechoslovakia” OR “Côte d'Ivoire” OR “Ivory Coast” OR “Côte dIvoire” OR “Djibouti” OR “Dominica” OR “Dominican Republic” OR “Ecuador” OR “Egypt” OR “El Salvador” OR “Equatorial Guinea” OR “Eritrea” OR “Estonia” OR “Ethiopia” OR “Fiji” OR “Gabon” OR “Gambia” OR “Georgia” OR “Ghana” OR “Gibraltar” OR “Greece” OR “Grenada” OR “Guam” OR “Guatemala” OR “Guinea” OR “Guinea-Bissau” OR “Guyana” OR “Haiti” OR “Honduras” OR “Hungary” OR “India” OR “Indonesia” OR “Iran” OR “Iraq” OR “Isle of Man” OR “Jamaica” OR “Jordan” OR “Kazakhstan” OR “Kenya” OR “Kiribati” OR “Korea” OR “Kosovo” OR “Kyrgyz Republic” OR “Kyrgyzstan” OR “Kirgizstan” OR “Lao PDR” OR “Laos” OR “Latvia” OR “Lebanon” OR “Lesotho” OR “Liberia” OR “Libya” OR “Lithuania” OR “Macao” OR “Macedonia” OR “Madagascar” OR “Malawi” OR “Malaysia” OR “Maldives” OR “Mali” OR “Malta” OR “Marshall Islands” OR “Mauritania” OR “Mauritius” OR “Mayotte” OR “Mexico” OR “Micronesia” OR “Moldova” OR “Mongolia” OR “Montenegro” OR “Morocco” OR “Mozambique” OR “Myanmar” OR “Namibia” OR “Nepal” OR “Netherlands Antilles” OR “New Caledonia” OR “Nicaragua” OR “Niger” OR “Nigeria” OR “Mariana Islands” OR “Oman” OR “Pakistan” OR “Palau” OR “Panama” OR “Papua New Guinea” OR “Paraguay” OR “Peru” OR “Philippines” OR “Poland” OR “Portugal” OR “Puerto Rico” OR “Romania” OR “Russian Federation” OR “Russia” OR “Rwanda” OR “Samoa” OR “Saudi Arabia” OR “Senegal” OR “Serbia” OR “Seychelles” OR “Sierra Leone” OR “Slovak Republic” OR “Slovakia” OR “Slovenia” OR “Solomon Islands” OR “Somalia” OR “South Africa” OR “South Sudan” OR “Sri Lanka” OR “Kitts and Nevis” OR “St Kitts” OR “Saint Kitts” OR “St Lucia” OR “Saint Lucia” OR “Grenadines” OR “Sudan” OR “Suriname” OR “Swaziland” OR “Syrian Arab Republic” OR “Syria” OR “São Tomé” OR “Tajikistan” OR “Tanzania” OR “Thailand” OR “Timor-Leste” OR “Togo” OR “Tonga” OR “Trinidad” OR “Tobago” OR “Tunisia” OR “Turkey” OR “Turkmenistan” OR “Tuvalu” OR “USSR” OR “Uganda” OR “Ukraine” OR “Uruguay” OR “Uzbekistan” OR “Vanuatu” OR “Venezuela” OR “Vietnam” OR “West Bank” OR “Gaza” OR “Yemen” OR “Yugoslavia” OR “Zambia” OR “Zimbabwe”) *OR* **TOPIC:** (((“developing” OR “less developed” OR “under developed” OR “underdeveloped” OR “transitional” OR “middle income” OR “low income” OR “lower income”) AND (“countr*” OR “nation” OR “nations” OR “world” OR “economy” OR “economies”)) OR ((“low” OR “lower”) AND (“gdp” OR “gnp” OR “gross domestic” OR “gross national”)) OR “lmic” OR “lmics” OR “Lamic” OR “lamics” OR “third world” OR “lami countries” OR “lami country”)  *Timespan=All years*  *Search language=Auto* |

1. **Cochrane Library** (Wiley)

Run on April 28, 2017

| ID | Search | Hits |
| --- | --- | --- |
| #1 | ((developing or "less developed" or "under developed" or underdeveloped or transitional or "middle income" or "low income" or "lower income") and (countr* or nation or nations or world or economy or economies)) or ((low or lower) and (gdp or gnp or "gross domestic" or "gross national")) or lmic or lmics or Lamic or lamics or third world or "lami countries" or "lami country" or Afghanistan or Albania or Algeria or "American Samoa" or Angola or Antigua and Barbuda or Argentina or Armenia or Aruba or Azerbaijan or Bahrain or Bangladesh or Barbados or Belarus or Belize or Benin or Bhutan or Bolivia or Bosnia and Herzegovina or Botswana or Brazil or Bulgaria or "Burkina Faso" or Burundi or "Cabo Verde" or "Cape Verde" or Cambodia or Cameroon or "Central African Republic" or Chad or Chile or China or Colombia or Comoros or Congo or "Costa Rica" or Croatia or Cuba or Cyprus or "Czech Republic" or Czechoslovakia or "Côte d'Ivoire" or "Ivory Coast" or "Côte dIvoire" or Djibouti or Dominica or "Dominican Republic" or Ecuador or Egypt or "El Salvador" or "Equatorial Guinea" or Eritrea or Estonia or Ethiopia or Fiji or Gabon or Gambia or Georgia or Ghana or Gibraltar or Greece or Grenada or Guam or Guatemala or Guinea or "Guinea-Bissau" or Guyana or Haiti or Honduras or Hungary or India or Indonesia or Iran or Iraq or "Isle of Man" or Jamaica or Jordan or Kazakhstan or Kenya or Kiribati or Korea or Kosovo or "Kyrgyz Republic" or Kyrgyzstan or Kirgizstan or "Lao PDR" or Laos or Latvia or Lebanon or Lesotho or Liberia or Libya or Lithuania or Macao or Macedonia or Madagascar or Malawi or Malaysia or Maldives or Mali or Malta or "Marshall Islands" or Mauritania or Mauritius or Mayotte or Mexico or Micronesia or Moldova or Mongolia or Montenegro or Morocco or Mozambique or Myanmar or Namibia or Nepal or "Netherlands Antilles" or "New Caledonia" or Nicaragua or Niger or Nigeria or "Mariana Islands" or Oman or Pakistan or Palau or Panama or Papua New Guinea or Paraguay or Peru or Philippines or Poland or Portugal or Puerto Rico or Romania or Russian Federation or Russia or Rwanda or Samoa or "Saudi Arabia" or Senegal or Serbia or Seychelles or "Sierra Leone" or "Slovak Republic" or Slovakia or Slovenia or "Solomon Islands" or Somalia or "South Africa" or "South Sudan" or "Sri Lanka" or "Kitts and Nevis" or "St Kitts" or "Saint Kitts" or "St Lucia" or "Saint Lucia" or Grenadines or Sudan or Suriname or Swaziland or "Syrian Arab Republic" or Syria or "São Tomé" or Tajikistan or Tanzania or Thailand or "Timor-Leste" or Togo or Tonga or Trinidad or Tobago or Tunisia or Turkey or Turkmenistan or Tuvalu or USSR or Uganda or Ukraine or Uruguay or Uzbekistan or Vanuatu or Venezuela or Vietnam or "West Bank" or Gaza or Yemen or Yugoslavia or Zambia or Zimbabwe:ti,ab,kw (Word variations have been searched) | 53683 |
| #2 | Integrat* or collaborat* or "Managed care" or "Care management" or "Shared care" or "Transmural care" or "Continuity of Patient Care" or "Continuity of Care" or "Case Management" or "Patient centered" or "Patient centred" or "People centered" or "People centred" or "Transitional care" or "Care transition*" or "Behavioral health home*" or "Behavioural health home*" or "Mental health home*" or "Primary care" or "Community health" or "Community based care" or "Community based health*" or "Community based mental health" or "Community based management" or "Primary health care" or "Barefoot doctor*" or "Village health worker*" or "Home care" or "Lay health" or (colocat* and (care or service*)):ti,ab,kw (Word variations have been searched) | 40671 |
| #3 | Depression or Depressed or Depressive or Mood or "Psychological indicator" or "Emotional functioning" or "Mental functioning" or "Psychological status" or "Psychological coping" or "Binge drink*" or Alcoholic* or Alcoholism or "Alcohol use disorder" or "Alcohol misuse" or "Alcohol abuse" or "Alcohol dependence" or "Alcohol addiction" or "Ethanol use disorder" or "Ethanol misuse" or "Ethanol abuse" or "Ethanol dependence" or "Ethanol addiction" or AOD or "Underage drink*" or "Under-age drink*":ti,ab,kw (Word variations have been searched) | 66097 |
| #4 | #1 and #2 and #3 Publication Year from 1990 to 2017 | 376 |

**Cochrane Database of Systematic Reviews: Issue 4 of 12, April 2017 – 31 results**

**Cochrane Central Register of Controlled Trials: Issue 3 of 12, March 2017 – 339 results**

**Cochrane Methodology Register: Issue 3 of 4, July 2012 – 2 results**

**NHS Economic Evaluation Database: Issue 2 of 4, April 2015** – **4 results**

1. **PsycINFO** (ProQuest)

Run on April 28, 2017

(SU.EXACT.EXPLODE("Developing Countries") OR ti((("developing" OR "less developed" OR "under developed" OR "underdeveloped" OR "transitional" OR "middle income" OR "low income" OR "lower income") AND ("countr*" OR "nation" OR "nations" OR "world" OR "economy" OR "economies")) OR (("low" OR "lower") AND ("gdp" OR "gnp" OR "gross domestic" OR "gross national")) OR "lmic" OR "lmics" OR "Lamic" OR "lamics" OR "third world" OR "lami countries" OR "lami country" OR "Afghanistan" OR "Albania" OR "Algeria" OR "American Samoa" OR "Angola" OR "Antigua" AND "Barbuda" OR "Argentina" OR "Armenia" OR "Aruba" OR "Azerbaijan" OR "Bahrain" OR "Bangladesh" OR "Barbados" OR "Belarus" OR "Belize" OR "Benin" OR "Bhutan" OR "Bolivia" OR "Bosnia" AND "Herzegovina" OR "Botswana" OR "Brazil" OR "Bulgaria" OR "Burkina Faso" OR "Burundi" OR "Cabo Verde" OR "Cape Verde" OR Cambodia OR Cameroon OR "Central African Republic" OR "Chad" OR "Chile" OR "China" OR "Colombia" OR "Comoros" OR "Congo" OR "Costa Rica" OR "Croatia" OR "Cuba" OR "Cyprus" OR "Czech Republic" OR "Czechoslovakia" OR "Côte d'Ivoire" OR "Ivory Coast" OR "Côte dIvoire" OR "Djibouti" OR "Dominica" OR "Dominican Republic" OR "Ecuador" OR "Egypt" OR "El Salvador" OR "Equatorial Guinea" OR "Eritrea" OR "Estonia" OR "Ethiopia" OR "Fiji" OR "Gabon" OR "Gambia" OR "Georgia" OR "Ghana" OR "Gibraltar" OR "Greece" OR "Grenada" OR "Guam" OR "Guatemala" OR "Guinea" OR "Guinea-Bissau" OR "Guyana" OR "Haiti" OR "Honduras" OR "Hungary" OR "India" OR "Indonesia" OR "Iran" OR "Iraq" OR "Isle of Man" OR "Jamaica" OR "Jordan" OR "Kazakhstan" OR "Kenya" OR "Kiribati" OR "Korea" OR "Kosovo" OR "Kyrgyz Republic" OR "Kyrgyzstan" OR "Kirgizstan" OR "Lao PDR" OR "Laos" OR "Latvia" OR "Lebanon" OR "Lesotho" OR "Liberia" OR "Libya" OR "Lithuania" OR "Macao" OR "Macedonia" OR "Madagascar" OR "Malawi" OR "Malaysia" OR "Maldives" OR "Mali" OR "Malta" OR "Marshall Islands" OR "Mauritania" OR "Mauritius" OR "Mayotte" OR "Mexico" OR "Micronesia" OR "Moldova" OR "Mongolia" OR "Montenegro" OR "Morocco" OR "Mozambique" OR "Myanmar" OR "Namibia" OR "Nepal" OR "Netherlands Antilles" OR "New Caledonia" OR "Nicaragua" OR "Niger" OR "Nigeria" OR "Mariana Islands" OR "Oman" OR "Pakistan" OR "Palau" OR "Panama" OR "Papua New Guinea" OR "Paraguay" OR "Peru" OR "Philippines" OR "Poland" OR "Portugal" OR "Puerto Rico" OR "Romania" OR "Russian Federation" OR "Russia" OR "Rwanda" OR "Samoa" OR "Saudi Arabia" OR "Senegal" OR "Serbia" OR "Seychelles" OR "Sierra Leone" OR "Slovak Republic" OR "Slovakia" OR "Slovenia" OR "Solomon Islands" OR "Somalia" OR "South Africa" OR "South Sudan" OR "Sri Lanka" OR "Kitts and Nevis" OR "St Kitts" OR "Saint Kitts" OR "St Lucia" OR "Saint Lucia" OR "Grenadines" OR "Sudan" OR "Suriname" OR "Swaziland" OR "Syrian Arab Republic" OR "Syria" OR "São Tomé" OR "Tajikistan" OR "Tanzania" OR "Thailand" OR "Timor-Leste" OR "Togo" OR "Tonga" OR "Trinidad" OR "Tobago" OR "Tunisia" OR "Turkey" OR "Turkmenistan" OR "Tuvalu" OR "USSR" OR "Uganda" OR "Ukraine" OR "Uruguay" OR "Uzbekistan" OR "Vanuatu" OR "Venezuela" OR "Vietnam" OR "West Bank" OR "Gaza" OR "Yemen" OR "Yugoslavia" OR "Zambia" OR "Zimbabwe") OR ab((("developing" OR "less developed" OR "under developed" OR "underdeveloped" OR "transitional" OR "middle income" OR "low income" OR "lower income") AND ("countr*" OR "nation" OR "nations" OR "world" OR "economy" OR "economies")) OR (("low" OR "lower") AND ("gdp" OR "gnp" OR "gross domestic" OR "gross national")) OR "lmic" OR "lmics" OR "Lamic" OR "lamics" OR "third world" OR "lami countries" OR "lami country" OR "Afghanistan" OR "Albania" OR "Algeria" OR "American Samoa" OR "Angola" OR "Antigua" AND "Barbuda" OR "Argentina" OR "Armenia" OR "Aruba" OR "Azerbaijan" OR "Bahrain" OR "Bangladesh" OR "Barbados" OR "Belarus" OR "Belize" OR "Benin" OR "Bhutan" OR "Bolivia" OR "Bosnia" AND "Herzegovina" OR "Botswana" OR "Brazil" OR "Bulgaria" OR "Burkina Faso" OR "Burundi" OR "Cabo Verde" OR "Cape Verde" OR Cambodia OR Cameroon OR "Central African Republic" OR "Chad" OR "Chile" OR "China" OR "Colombia" OR "Comoros" OR "Congo" OR "Costa Rica" OR "Croatia" OR "Cuba" OR "Cyprus" OR "Czech Republic" OR "Czechoslovakia" OR "Côte d'Ivoire" OR "Ivory Coast" OR "Côte dIvoire" OR "Djibouti" OR "Dominica" OR "Dominican Republic" OR "Ecuador" OR "Egypt" OR "El Salvador" OR "Equatorial Guinea" OR "Eritrea" OR "Estonia" OR "Ethiopia" OR "Fiji" OR "Gabon" OR "Gambia" OR "Georgia" OR "Ghana" OR "Gibraltar" OR "Greece" OR "Grenada" OR "Guam" OR "Guatemala" OR "Guinea" OR "Guinea-Bissau" OR "Guyana" OR "Haiti" OR "Honduras" OR "Hungary" OR "India" OR "Indonesia" OR "Iran" OR "Iraq" OR "Isle of Man" OR "Jamaica" OR "Jordan" OR "Kazakhstan" OR "Kenya" OR "Kiribati" OR "Korea" OR "Kosovo" OR "Kyrgyz Republic" OR "Kyrgyzstan" OR "Kirgizstan" OR "Lao PDR" OR "Laos" OR "Latvia" OR "Lebanon" OR "Lesotho" OR "Liberia" OR "Libya" OR "Lithuania" OR "Macao" OR "Macedonia" OR "Madagascar" OR "Malawi" OR "Malaysia" OR "Maldives" OR "Mali" OR "Malta" OR "Marshall Islands" OR "Mauritania" OR "Mauritius" OR "Mayotte" OR "Mexico" OR "Micronesia" OR "Moldova" OR "Mongolia" OR "Montenegro" OR "Morocco" OR "Mozambique" OR "Myanmar" OR "Namibia" OR "Nepal" OR "Netherlands Antilles" OR "New Caledonia" OR "Nicaragua" OR "Niger" OR "Nigeria" OR "Mariana Islands" OR "Oman" OR "Pakistan" OR "Palau" OR "Panama" OR "Papua New Guinea" OR "Paraguay" OR "Peru" OR "Philippines" OR "Poland" OR "Portugal" OR "Puerto Rico" OR "Romania" OR "Russian Federation" OR "Russia" OR "Rwanda" OR "Samoa" OR "Saudi Arabia" OR "Senegal" OR "Serbia" OR "Seychelles" OR "Sierra Leone" OR "Slovak Republic" OR "Slovakia" OR "Slovenia" OR "Solomon Islands" OR "Somalia" OR "South Africa" OR "South Sudan" OR "Sri Lanka" OR "Kitts and Nevis" OR "St Kitts" OR "Saint Kitts" OR "St Lucia" OR "Saint Lucia" OR "Grenadines" OR "Sudan" OR "Suriname" OR "Swaziland" OR "Syrian Arab Republic" OR "Syria" OR "São Tomé" OR "Tajikistan" OR "Tanzania" OR "Thailand" OR "Timor-Leste" OR "Togo" OR "Tonga" OR "Trinidad" OR "Tobago" OR "Tunisia" OR "Turkey" OR "Turkmenistan" OR "Tuvalu" OR "USSR" OR "Uganda" OR "Ukraine" OR "Uruguay" OR "Uzbekistan" OR "Vanuatu" OR "Venezuela" OR "Vietnam" OR "West Bank" OR "Gaza" OR "Yemen" OR "Yugoslavia" OR "Zambia" OR "Zimbabwe") AND pd(>19900101))

**AND**

((SU.EXACT("Continuum of Care") OR SU.EXACT("Case Management") OR SU.EXACT.EXPLODE("Community Mental Health Services") OR SU.EXACT("Primary Health Care") OR SU.EXACT("Integrated Services") OR SU.EXACT("Home Care")) OR ti("Integrat*" OR "Collaborat*" OR "Managed care" OR "Care management" OR "Shared care" OR "Transmural care" OR "Continuity of Patient Care" OR "Continuity of Care" OR "Case Management" OR "Patient centered" OR "Patient centred" OR "People centered" OR "People centred" OR "Transitional care" OR "Care transition*" OR "Behavioral health home*" OR "Behavioural health home*" OR "Mental health home*" OR "Primary care" OR "Community health" OR "Community based care" OR "Community based health*" OR "Community based mental health" OR "Community based management" OR "Primary health care" OR "Barefoot doctor*" OR "Village health worker*" OR "Home care" OR "Lay health" OR ("colocat*" W4 ("care" OR "service*"))) OR ab("Integrat*" OR "Collaborat*" OR "Managed care" OR "Care management" OR "Shared care" OR "Transmural care" OR "Continuity of Patient Care" OR "Continuity of Care" OR "Case Management" OR "Patient centered" OR "Patient centred" OR "People centered" OR "People centred" OR "Transitional care" OR "Care transition*" OR "Behavioral health home*" OR "Behavioural health home*" OR "Mental health home*" OR "Primary care" OR "Community health" OR "Community based care" OR "Community based health*" OR "Community based mental health" OR "Community based management" OR "Primary health care" OR "Barefoot doctor*" OR "Village health worker*" OR "Home care" OR "Lay health" OR ("colocat*" W4 ("care" OR "service*"))) AND pd(>19900101))

**AND**

((SU.EXACT.EXPLODE("Major Depression") OR SU.EXACT.EXPLODE("Alcohol Abuse") OR SU.EXACT("Alcohol Drinking Patterns")) OR ti("Depression" OR "Depressed" OR "Depressive" OR "Mood" OR "Psychological indicator" OR "Emotional functioning" OR "Mental functioning" OR "Psychological status" OR "Psychological coping" OR "Binge drink*" OR "Alcoholic*" OR "Alcoholism" OR "Alcohol use disorder" OR "Alcohol misuse" OR "Alcohol abuse" OR "Alcohol dependence" OR "Alcohol addiction" OR "Ethanol use disorder" OR "Ethanol misuse" OR "Ethanol abuse" OR "Ethanol dependence" OR "Ethanol addiction" OR "AOD" OR "Underage drink*" OR "Under-age drink*") OR ab("Depression" OR "Depressed" OR "Depressive" OR "Mood" OR "Psychological indicator" OR "Emotional functioning" OR "Mental functioning" OR "Psychological status" OR "Psychological coping" OR "Binge drink*" OR "Alcoholic*" OR "Alcoholism" OR "Alcohol use disorder" OR "Alcohol misuse" OR "Alcohol abuse" OR "Alcohol dependence" OR "Alcohol addiction" OR "Ethanol use disorder" OR "Ethanol misuse" OR "Ethanol abuse" OR "Ethanol dependence" OR "Ethanol addiction" OR "AOD" OR "Underage drink*" OR "Under-age drink*") AND pd(>19900101))

Results = 1241

1. **WHO Global Index Medicus** (www. globalhealthlibrary.net/)

Databases searched: LILACS (Americas), WPRIM (Western Pacific), IMEMR (Eastern Mediterranean), IMSEAR (Southeast Asia), AIM (Africa)

Run on April 28, 2017

(tw:(((Integrated OR integration OR collaborate OR collaborative OR "Managed care" OR "Care management" OR "Shared care" OR "Transmural care" OR "Continuity of Patient Care" OR "Continuity of Care" OR "Case Management" OR "Patient centered" OR "Patient centred" OR "People centered" OR "People centred" OR "Transitional care" OR "Care transition" OR "care transitions" OR "Behavioral health home" OR "Behavioral health home" OR "Behavioural health homes" OR "Mental health home" OR "Mental health homes" OR "Primary care" OR "Community health" OR "Community based care" OR "Community based health" OR "Community based healthcare" OR "Community based mental health" OR "Community based management" OR "Primary health care" OR "Barefoot doctor" OR "Barefoot doctors" OR "Village health worker" OR "Village health workers" OR "Home care" OR "Lay health" )) ))

**AND**

(tw:(((Depression OR Depressed OR Depressive OR Mood OR "Psychological indicator" OR "Emotional functioning" OR "Mental functioning" OR "Psychological status" OR "Psychological coping" OR "Binge drink" OR "Binge drinking" OR Alcoholic OR Alcoholics OR Alcoholism OR "Alcohol use disorder" OR "Alcohol misuse" OR "Alcohol abuse" OR "Alcohol dependence" OR "Alcohol addiction" OR "Ethanol use disorder" OR "Ethanol misuse" OR "Ethanol abuse" OR "Ethanol dependence" OR "Ethanol addiction" OR AOD OR "Underage drink" OR "Underage drink" OR "Under-age drinking"))))

Results = 1254
